# Functional brain activations correlated with association strength and prediction error during novel symbol–speech sound learning

**DOI:** 10.1162/imag_a_00439

**Published:** 2025-01-13

**Authors:** Gorka Fraga-González, Patrick Haller, David Willinger, Vanessa Gehrig, Nada Frei, Silvia Brem

**Affiliations:** Neurolinguistics and Department of Psychology, University of Zurich, Zurich, Switzerland; Department of Child and Adolescent Psychiatry and Psychotherapy, University Hospital of Psychiatry, University of Zurich, Zurich, Switzerland; Department of Computational Linguistics, University of Zurich, Zurich, Switzerland; Neuroscience Center Zurich, University of Zurich and ETH Zurich, Zurich, Switzerland; Department of Psychology and Psychodynamics, Karl Landsteiner University of Health Sciences, Krems an der Donau, Austria; MR-Center of the Department of Psychiatry, Psychotherapy and Psychosomatics and the Department of Child and Adolescent Psychiatry and Psychotherapy, Psychiatric Hospital, University of Zurich, Zurich, Switzerland; University Research Priority Program (URPP), Adaptive Brain Circuits in Development and Learning (AdaBD), University of Zurich, Zurich, Switzerland

**Keywords:** audiovisual learning, letter–speech sound, model-based fMRI, reinforcement learning drift diffusion model

## Abstract

Efficient learning of letters–speech sound associations results in the specialization of visual and audiovisual brain regions, which is crucial for the development of proficient reading skills. However, the brain dynamics underlying this learning process remain poorly understood, and the involvement of learning and performance monitoring networks remains underexplored. Here we applied two mutually dependent feedback learning tasks in which novel symbol–speech sound associations were learned by 39 healthy adults. We employed functional magnetic resonance (fMRI) along with a reinforcement learning drift diffusion model to characterize trial-by-trial learning in behavior and brain. The model-based analysis showed that posterior–occipital activations during stimulus processing were positively modulated by trial-wise learning, as indicated by the increase in association strength between audiovisual pairs. Prediction errors, describing the update mechanism to learn from feedback across trials, modulated activations in several mid-frontal, striatal, and cingulate regions. Both tasks yielded similar patterns of results, despite differences in their relative difficulty. This study elucidates the processes involved in audiovisual learning that contribute to rapid visual specialization within a single experimental session and delineates a set of coactivated regions involved in learning from feedback. Our paradigm provides a framework to advance our understanding of the neurobiology of learning and reading development.

## 1 Introduction

Learning to map symbols to speech sounds is an important milestone in learning to read alphabetic orthographies. This process leads to reorganization of visual and multisensory integration areas when learning how to read (Hervais-Adelman et al., 2019;van Atteveldt & Ansari, 2014). Brain activity in these areas correlates with reading skills and developmental dyslexia has been associated with atypical development and, mainly, reduced activation in these regions compared with unimpaired readers (Norton et al., 2015;Richlan, 2014,^2019^). A crucial aspect that remains unclear is which other learning-related systems contribute to the development of an efficient reading network during the acquisition of reading skills (Fraga-González et al., 2017). For example, there is evidence suggesting a potential involvement of frontostriatal circuits in reading disorders (Hancock et al., 2017;Krishnan et al., 2016;Nicolson & Fawcett, 2007, but seeCignetti et al., 2020). A deeper understanding of these contributions will be an important step forward in characterizing the neurobiology of reading and learning disorders.

Previous neuroimaging studies in this context have examined how children develop well-established letter–speech sound associations over months or years of instruction (Fraga-González et al., 2021;Maurer et al., 2005;Wang et al., 2020), or after trainings or interventions over several days or weeks (Brem et al., 2010;Di Pietro et al., 2023;Fraga-González et al., 2016;Pleisch et al., 2019;Žarić et al., 2015). A previous study on pre-reading children following a short training (<40 min) of artificial letters suggested that temporoparietal responses to the trained symbols could be used to predict future reading skills (Karipidis et al., 2018). The evidence from these studies demonstrates that visual and multisensory areas specialize after varying learning period durations and in different developmental stages. Importantly, several behavioral studies support the relation of this type of novel symbol–sound learning and different early reading and cognitive skills in kindergarteners (Horbach et al., 2015), 5-year olds (Altarelli et al., 2020), and adults (Pasqualotto et al., 2021). However, they still lack specificity in terms of cognitive mechanisms and additional brain systems supporting this specialization of visual and audiovisual areas (Bonte & Brem, 2024). In addition, one of the studies suggests a limited time window for detecting specialized responses (Fraga-González et al., 2021), which calls for a closer examination of learning dynamics within a narrower time frame.

In order to avoid over-learned and over-exposed stimuli, many studies in adults used artificial script learning tasks, and showed increased ventral occipitotemporal activations after a few hours (Brem et al., 2018) or within a few days of training (Hashimoto & Sakai, 2004;Perrone-Bertolotti et al., 2014;Xue et al., 2006). A recent study using magnetoencephalography (MEG) examined brain activations in adults when learning novel grapheme–phoneme associations and monitored performance during the learning block as well as 1 day after learning (Xu et al., 2020). The study included training blocks in which the audiovisual pairs were followed by learning cues, and test blocks in which participants had to respond whether a given pair was a match, no match, or unknown. Besides learnable pairs, there were control pairs for which the learning cues were not informative. The results showed changes in superior temporal and dorsal parietal sources with learning. In addition, middle and inferior temporal regions, possibly reflecting activation from regions such as insula or hippocampus, were engaged when using the cues to learn associations. This MEG study, although limited in its spatial resolution, provides an interesting window into rapid dynamics within early grapheme–phoneme learning.

Two previous EEG studies used a similar learning task to examine differences between typical and dyslexic adult readers in physiological responses to feedback (Fraga-González et al., 2019) and in oscillatory networks (Fraga-González et al., 2022). Their findings suggested differences between typical and impaired readers that would require further specification in terms of both brain networks and cognitive processes involved. In the current study, we used a feedback learning (FBL) paradigm adapted from those studies. The task required participants to learn associations between unfamiliar false fonts (i.e., letter-like symbols) and speech sounds based on the feedback presented on screen after a yes/no response. In this paradigm, learning occurs within the same block where participants respond, and brain activations related to learning novel audiovisual pairs are examined on a trial-by-trial basis within the experimental blocks (<8 min each). Our task was set to simulate an important part of learning an alphabetic script, where practice and trial errors and feedback allow the reader to establish new associations. In addition, we included an additional task that simulates the role of diacritic marks, common in orthographic languages, as well as the inconsistencies between phonemes and graphemes, as in opaque orthographies like English. This additional task (FBL-B) follows the same principles and depends upon the stimuli from the main task (FBL-A), since diacritic marks modify the speech sounds associated with the false fonts from the preceding FBL-A blocks. The additional goal of task B, which is expected to be more demanding, was as well to generate an additional level of difficulty and variability in performance. The adult population in this study was also chosen to allow flexibility in length and complexity of the paradigm design, which is important for our goal of finding new neural and behavioral descriptors of individual variability in learning.

The previous work fromFraga-González et al. (2019,^2022^) lacked specificity in describing the brain areas involved in this task. The current functional magnetic resonance imaging (fMRI) environment allows a spatially resolved network characterization not possible with electrophysiological recordings. Here, the frontostriatal circuits are of special interest. Activity in the anterior cingulate cortex (ACC) has been associated with a variety of functions relevant to our task, like feedback/reinforcement learning, error detection, action selection, and conflict monitoring (see review inHolroyd & Yeung, 2011). A recent review argues for three core computational principles in the ACC: hierarchical decision making, spatiotemporal models of the environment, and cost evaluation (Holroyd & Verguts, 2021). In this context, an important concept is the prediction error (PE), that is the discrepancy between expectations and outcomes, proposed to drive learning (Friston, 2010). The ACC is involved in adjusting responses and decision making using this predictive signal together with the surrounding prefrontal cortex (Alexander & Brown, 2019). The regions involved in encoding these PE signals are striatal regions such as caudate and putamen (Garrison et al., 2013;Schultz, 2013), which are also linked to different forms of motor, instrumental, and associative learning (Brovelli et al., 2011;Liljeholm & O’Doherty, 2012).

In the context of reading acquisition, prediction error and the more general*predictive coding*scheme for describing brain function are central to an interactive account on the specialization of visual occipitotemporal regions for reading. Predictive coding is a framework that has been used to describe perception in different contexts and to denominate multiple information processing algorithms (Spratling, 2017). It can generally describe the interaction between bottom-up encoding (e.g., of sensory input) and top-down predictions (e.g., from previous knowledge). In the interactive account byPrice and Devlin (2011), this framework is used in a broader multisensory context. They propose that the ventral occipitotemporal (vOT) region could serve as an interface between visual sensory input in occipital areas and higher-level regions, integrating predictions from phonological and semantic information. Accordingly, the prediction signals would result in higher activation of vOT areas during the initial stages of learning, when prediction error signals are high. As expertise increases and prediction errors decrease, these activations would be reduced. The current study builds on this conceptualization of the predictive coding framework, considering prediction error as a signal of surprise that arises from discrepancies between anticipated and received feedback. In the present analysis, audiovisual learning is represented by the model parameter association strength, which is updated on each trial based on the prediction error (PE) generated by processing the feedback following each response. In our task, phonological information is associated with visual symbols as the task progresses, leading to the expectation that this information will contribute to prediction and prediction error signals that can influence activity in the ventral occipitotemporal (vOT) areas.

Besides spatial specificity, the preceding work byFraga-González et al. (2019,^2022^) with the previous version of this FBL task also lacks specificity in terms of the cognitive processes that may explain differences in task performance. In the current analysis, we expand beyond the conventional measures of performance (accuracy and reaction times) by applying a computational model approach. In order to describe learning components related to the frontostriatal network and associative learning, our experimental task was analyzed with a reinforcement learning drift diffusion model (RLDDM). The reinforcement learning part of this model is interesting as it allows describing trial-by-trial learning, which in the present context reflects the mapping of symbols to speech sounds. The drift diffusion part of the model adds the global dynamics of decision making in terms of speed/accuracy trade-offs (Pedersen et al., 2017). According to a previous report on children with dyslexia, these decision processes in a context of uncertainty could partially explain some of the deficits in dyslexics’ performance (Zeguers et al., 2011). Thus, the choice of this model broadens the cognitive descriptors derived from the task performance, and makes it an interesting tool to search for novel markers that could ultimately help characterizing clinical populations. The model-based cognitive neuroscience approach in this analysis is intended to capture underlying cognitive processes and their associated brain activation patterns which may be overlooked in basic reaction times and accuracy analysis (Forstmann & Wagenmakers, 2015).

In summary, the primary goal of the current study is to characterize changes in brain function during trial-by-trial learning of symbol–speech sound associations, as described by the parameters of a RLDDM. We focus on examining how learning new audiovisual associations modulates brain activations in visual and audiovisual regions, as well as in regions involved in associative learning and feedback processing. As a secondary aim, the current paradigm investigates individual differences in learning that could be linked to cognitive performance and reading skills. Ultimately, the broader goal of this research is to identify new neurocognitive markers to predict and characterize both typical and atypical reading development.

## 2 Methods

### 2.1 Participants

The current study is based on a sample of 39 healthy adult participants (21 females; age 25.19 ± 3.12 years [range 18.14–32.51], seeAppendix Table A1for details). Participants were recruited using university platforms and social media to be right-handed (Swiss-) German-speaking, and between the age of 18 and 35 years. They had on average 16.38 ± 2.65 [range 12–25] years of education. They were screened for contraindications for MRI (e.g., metallic implants, neurostimulators, or cardiac pacemakers, pregnancy) and neurological disorders. Two of participants in the sample reported attentional problems and problems with spelling and reading but no diagnosis of psychiatric disorders. The current study sample was obtained from an initial pool of 43 participants from which 1 participant was excluded due to voluntary interruption of the scanning, 1 was excluded due to technical problems during scanning, 1 participant did not comply to the experimental task instructions, and 1 participant could not be scanned due to MR contraindications. A further exclusion criterion was a nonverbal IQ < 80 in the cognitive assessments, not fulfilled by any participant in this analysis. Additionally, participants filled in an adult reading history questionnaire (Lefly & Pennington, 2000) on their reading history and habits. The project was approved by the local ethics committee of the Canton of Zurich in Switzerland (BASEC-No. 2019-02296) and was performed in accordance with the Declaration of Helsinki. Participants signed a written informed consent form before participating in the study.

Regarding statistical power of the current sample, the hierarchical Bayesian methods to estimate the model in this study have been proposed to enhance statistical power, requiring fewer data per subject/condition than other approaches (Wiecki et al., 2013).Wiecki et al. (2013)demonstrated an advantage of the hierarchical structure of the model to detect effects in different group sizes up to 28 participants. Despite this advantage, the current sample size is limited in its statistical power and generalizability (see Limitations).

### 2.2 Cognitive assessments

All participants performed a series of cognitive and reading tests. The descriptive statistics of performance are summarized inAppendix Table A1.

Due to COVID-19-related measures, some of the tests were conducted via video call using an application secured by the university (https://www.zi.uzh.ch/en/support/audio-video.html). The following tests were conducted online. Working memory was assessed with the backward and forward digit span subtest from the*Wechsler Adult Intelligence Scale-fourth edition*(WAIS-IV;Wechsler & Petermann, 2012). Rapid automated naming (RAN) was used as a measure of general naming speed with the object (animal) and color naming tasks from the*Test zur Erfassung der phonologischen Bewusstheit und der Benennungsgeschwindigkeit*(TEPHOBE;Mayer, 2011).

**Appendix Fig. A1.**
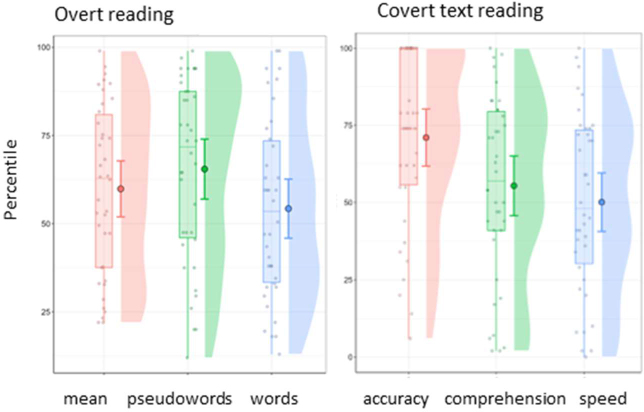
Percentile scores for overt reading scores (SLRT-II; left plot) and for covert, text reading accuracy, comprehension, and speed scores (LGVT; right plot).

In addition, the following tests were performed in the MR facilities before commencing the neuroimaging recordings. Overt word and pseudoword reading fluency was assessed with the*Salzburger Lese- und Rechtschreibtest*(SLRT-II;Moll & Landerl, 2010). The number of correctly and overtly read items in 1 min is used as main measure of this test. Since this is the main test to estimate reading abilities, the distributions of percentile scores are shown inAppendix Figure A1. Moreover, comprehension, velocity, and accuracy of covert text reading were assessed with the*Lesegeschwindigkeits- und Verständnistest für die Klassen 5-12+*(LGVT;Schneider et al., 2017). Participants are instructed to covertly read as fast and accurately as possible a brief text within 6 min. Within the text, there are several single-choice questions in which they must select one out of three words that fit the paragraph content. The percentile scores are presented inAppendix Figure A1. Spelling was tested with the*Rechtschreibetest- aktuelle Rechtschreiberegelung*(RST;Ibrahimović & Bulheller, 2013), in its short version for 14–60 years of age. In this test, participants get a transcript of a text with some gaps that they must fill after listening to the experimenter read the text aloud. To avoid inconsistencies between experimenters, we presented a video recording of a linguist reading the text. The nonverbal intelligence IQ was estimated with the*Reynolds intellectual assessment scales*(RIAS;Reynolds & Kamphaus, 2003).

### 2.3 Task and stimuli

The tasks performed in the MR scanner are an adaptation and extension of a task previously used in two electrophysiological studies (Fraga-González et al., 2019,^2022^). It was programmed and presented using Presentation^®^software (version 20.1,www.neurobs.com). The tasks are illustrated inFigure 1. Participants were instructed that they had to learn new symbol–speech sound mappings by deciding on each trial whether the audiovisually presented pair was correct or not, and then receiving feedback on the screen. In each trial, one symbol and one speech sound were presented simultaneously and participants were instructed to press the left or right button to indicate whether they thought the pair was a correct match or not. They were instructed to respond in every trial and to guess if unsure in order to learn the associations via feedback. The left/right button assignment to yes/no responses was counterbalanced across participants. The symbols remained on screen for the duration of the trials, which was terminated by the participant’s button press. After each response, the feedback provided was a happy or a sad smiley to indicate if the response was correct or incorrect, respectively. If no response was given within a 2,500 ms interval, a “Schneller” (“Faster”) feedback would be presented before the next trial to prevent too slow responses or inactivity. All feedback remained for a mean of 2,000 ms on screen (jittered durations drawn from a normal distribution of mean 2,000 ± SD = 500 ms). A fixation cross followed feedback until the next trial. The durations of this fixation were drawn from a normal distribution of 2,500 ± 500 ms. There were two learning blocks in each task, each block with 48 trials in which six different symbol–speech sounds could be learned. Each sound was repeated across eight trials, 50% of them showing the correct symbol and 50% showing the incorrect symbol. Pseudorandomized sequences were generated constraining the appearance of consecutive trials in which the same pair or sound was presented.

**Fig. 1.**
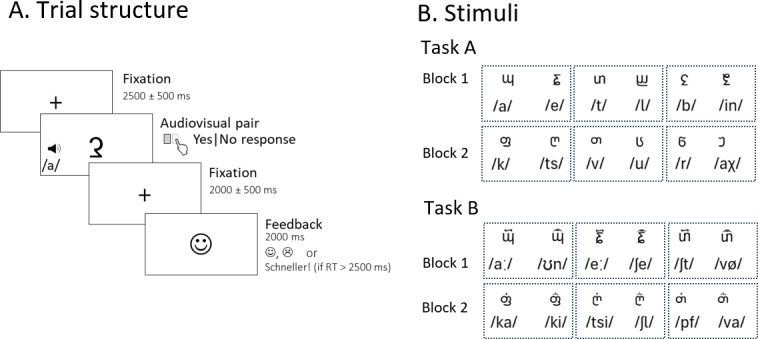
Task design. (A) Trial design in which a symbol and a phoneme are presented simultaneously, and response terminated. Feedback is presented for 2,000 ms and indicates whether the response is correct, incorrect, or too slow. (B) Table showing the visual stimuli and phonemes annotated in the International Phonetic Alphabet (IPA). Dashed lines indicate groups of stimuli presented together, with the assignment of correct pairs randomized across subjects. The first three symbols from task A appeared in task B with diacritic marks.

The phonemes (seeFig. 1B) were obtained from a pool of stimuli created in 2014 for different projects in our laboratory. They were recorded by professional (Swiss-) German radio speakers in a sound-proof recording booth at the UZH phonetics laboratory. Only the phonemes from the female speaker were used in this study. The audio files had a sampling rate of 44.1 kHz and 16 bits per sample. The files were normalized using the normalize function of Audacity^®^(version 2.3.0,www.audacityteam.org) which is a peak normalization effect that applies gains or reduction of the audio to adjust the peak to the desired level. The function was run with the options to remove direct current offset (centered on 0.0 vertically) and with the option to normalize peak amplitude to -0.5 db. Loudness checks were performed in the MRI environment to ensure they were presented at a sufficiently loud volume without resulting in discomfort to the participants. The same volume settings were applied to all participants, which were asked during the practice whether they could hear clearly the phonemes and whether they felt the loudness level was comfortable. The phonemes in task A had durations ranging from 0.459 to 0.913 s, mean 0.615 ± SD = 0.122 s. In task B, the range was 0.522 to 0.966 s and the mean and SD 0.701 ± 0.132 s. The result of a*t-test*comparing durations of phonemes between the tasks was*t*(21.85) = 1.65,*p*= 0.113).

The A and B tasks shared the same principles and design but varied in the stimuli presented (seeFig. 1B). They were presented always consecutively as the second task (B) builds on the stimuli learned in task A. In task FBL-A, the false fonts were single characters from a pseudofont. In the subsequent FBL-B, three of the false fonts from the same block in task A were presented with two different diacritic marks on top, leading to six new false fonts associated with new speech sounds. The speech sounds were either completely different, a prolongation, or a modification of those associated with the symbol in previous part. For example, the symbol N is paired with the speech sound /a/ in FBL-A and is presented as pairs P /aa/ and O /ao/ in FBL-B. The blocks in both parts started with a very brief practice sequence to ensure the participants understood the principle. In task A, this practice showed nine trials of three new false fonts and sounds that were not presented later in the block. In task B, the practice included six response-terminated trials to “refresh” the knowledge of the previously learned associations, by showing false fonts and sounds that would later be presented with the modifier marks.

### 2.4 Computational model

The basic analysis of task performance based on proportion of correct responses and reaction times (see Statistical Analysis) provides limited opportunities for interpretation on the underlying cognitive processes. Thus, we applied a computational model to derive more fine-grained parameters that can be later correlated with learning-related brain areas and with other cognitive skills. We chose a reinforcement learning drift diffusion model (RLDDM;Pedersen et al., 2017) as it has been proposed to reveal both learning and decision-making processes, which are both relevant to perform the task. There are several parameters from this model of special interest in our analyses. The parameter*association strength*from the reinforcement learning part of the model describes the process of mapping letters to speech sounds within the learning block. The main parameters of interest from the drift diffusion model,*drift rate*and*decision boundary*, describe how subjects accumulate information to make their responses, as well as trade-offs between speed and accuracy, respectively. In addition, the*nondecision time*parameter from the drift model disentangles the component from the reaction times that can be attributable to general processing speed rather than to a learning-based decision. The RLDDM is illustrated inFigure 2. The model parameters and priors are presented inAppendix Table A2.

**Fig. 2.**
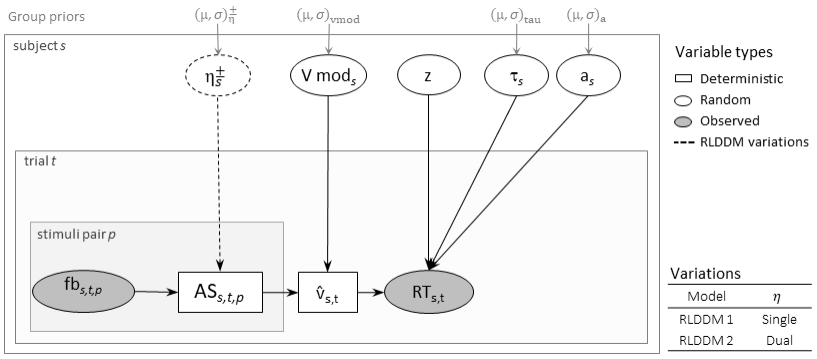
Schematic of the reinforcement learning drift diffusion model (RLDDM). Deterministic variables are illustrated with rectangular nodes, random variables with round nodes, and observed data with gray round nodes. Parameters are estimated in a hierarchical Bayesian framework. The subject parameters are estimated from group means*μ*and variance*σ*parameters. Learning rates (*η*) modulate the trial-by-trial associative strength (AS; or expected value) which are updated depending on the feedback. The mean AS modulated by the*v*mod free parameter is used to estimate the drift rate*v*per trial. Learning rates could be single or dual (for + and – prediction errors).*a*= decision boundary;*τ*= nondecision time;*z*= starting point (fixed parameter); fb = feedback;*s*= subject;*t*= trial;*p*= symbol–sound paired; v = drift rate.

The first component of the RLDDM is a reinforcement learning model (RL;Rescorla & Wagner, 1972). The basic principle of RL models is that learning is driven by unexpected outcomes, such as occurrence or absence of reward, which are captured by a prediction error, PE signal. The PE describes the difference between observed and predicted outcomes and is used to update expectations and adapt behavior in subsequent occurrences. This updating process is described byEquation (1)

$$AS_{t}=AS_{t-1}+\eta \;\;\cdot \;\;\left(f_{t}-AS_{t-1}\right)$$ (1)

where*t*refers to the current trial and*t*-1 the previous trial with the symbol in current trial. The formula describes how the association strength AS between the stimuli in a trial and its outcome is updated based on the differences between the feedback obtained*f*and the outcome expected from the previous occurrence. The term AS is used as equivalent to the expected values of an outcome (Machina, 1987). The trial-wise updating is modulated by a learning rate parameter*η*per subject, ranging from 0 to 1 and with larger values describing faster adaptation to grapheme–phoneme associations. In the RL models, the response choice is often modeled by using a softmax linking function (Luce, 1959) in which the probabilities on choosing a response over others are based on reward values. In the softmax case, the subject’s sensitivity to a reward is captured by a sensitivity parameter*β*, reflecting a subject’s individual importance of value differences. However, the softmax choice rule does not allow to take into account latency or differentiate between fast and accurate responses from those slow and conservative.

In the RLDDM, the softmax choice rule is replaced by a drift diffusion model (DDM;Ratcliff, 1978). The DDM families assume that a decision between two options is based on accumulating noisy evidence in favor for one of the options, until a decision threshold is reached. These models have been applied to data from many different psychological domains (see an overviewRatcliff et al., 2016). In brief, in the DDM, the distributions of accuracy and RTs depend on several parameters. The main parameter is the drift ratev, which indicates how fast the decision process reaches the boundaries (higher values mean faster and more accurate responses). The boundary separation or decision threshold parameteraadjusts how much noisy evidence is needed before reaching a threshold, that is, the speed–accuracy trade-off (higher values indicating slower and more accurate decisions). A starting pointzrepresents an initial bias toward a response. It was initially set to 0.5 (where*z*ranges from 0 to 1) because participants had no prior knowledge of the correct association, implying no initial bias toward any specific audiovisual pair. The nondecision timeτdescribes the time of encoding the stimuli and preparing the motor response. In the current RLDDM, the drift rate is described per trial$v_{t}$, as the scaled mean of the AS of the two reinforced options. SeeEquation (2):

$$v_{t}=v_{mod}\cdot \left(\frac{AS_{cor,t}+AS_{inc,t}}{2}\right).$$ (2)

In our study, the expected values are defined as the AS of correct or incorrect responses. The scaling factor$v_{mod}$is a free parameter, similar to the inverse temperatureβin the softmax rule, which describes the degree of sensitivity, that is, how much the choice is conditioned by association strengths. In the current analysis, the mean of the association strengths is used, since drift rate is expected to be highest when both associations are well known, medium when only one is well known, and lowest when none of the associations has been learned. The RTs and accuracy are simultaneously estimated in RLDDM, as in the standard DDM, using the Wiener first passage time (WFPT) distribution. This distribution describes the likelihood of an observing a response and RT and uses the DDM parameters described above:Equation (3)

$$\left(D_{t},RT_{t}\right)\sim \text{WFPT}\left(a,\tau ,z,v_{t}\right)$$ (3)

#### 2.4.1 Model fitting and model comparisons

**Appendix Fig. A2.**
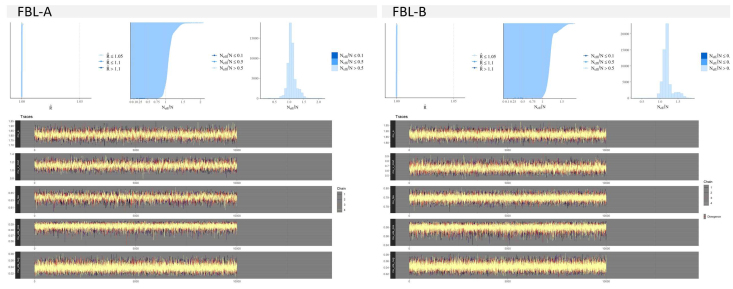
Model diagnostics for the RLDDM-2 (model with dual learning rates) with 4 chains of 10,000 iterations (+4,000 burn-in) for tasks A and B. Top panels$\hat{R}$convergence estimate (left) and ratios of effective sample size to total sample size (middle and right). Bottom plots are trace plots of the four chains for the group parameters.

Parameters were estimated using a Bayesian hierarchical modeling approach, which has been suggested to be advantageous for parameter estimation with limited amount of data (Pedersen & Frank, 2020;Vandekerckhove et al., 2011;Wiecki et al., 2013). We used the Markov Chain Monte Carlo methods implemented in Stan (Carpenter et al., 2017) with the interface to Stan, the “cmdstan” package (version 2.25.0;Stan Development Team., 2021). We ran 4 chains of 10,000 iterations each with 4,000 warm-up samples. Weakly informative priors were both modeled (seeAppendix Table A2). Convergence was assessed with the Gelman–Rubin convergence diagnostic$\hat{R}$(Gelman et al., 1995). Values of$\hat{R}$≤ 1.01 are considered indicative of successful convergence; this criterion was fulfilled by our parameters (see trace plots of chains and convergence diagnostics inAppendix Fig. A2).

In the current study, we run two model variations: one model with separate learning rates*ηs*for positive and negative PE, and one model with a single learning rate (Pedersen et al., 2017, see model illustration inFig. 2). We compared the models using the Widely Applicable Information Criterion (WAIC;Watanabe, 2010). The WAIC is computed from the log pointwise predictive density using the variance of individual terms summed over the data points to correct for model complexity. The prior distributions from which the different group and subject level parameters were drawn are presented inAppendix Table A2.

### 2.5 MR data acquisition and preprocessing

Participants took part in one experimental session lasting approximately 2.5 h. Before entering the scanner, they underwent behavioral assessments (see Cognitive Assessments), completed the MR standard safety screening, and received detailed instructions about the session and the current task. During the scanning session, the learning task and anatomical scan were both preceded and followed by a short visual target detection task, which is not analyzed in this study. MRI data were recorded on a Philips Achieva 3 Tesla scanner (Best, The Netherlands) using a 32-element receive head coil. Using a T2-weighted whole-brain gradient-echo echo planar image sequence, 460 volumes were acquired for each experimental block [Slices = 32; repetition time = 1,000 s; echo time = 30 ms; slice gap = 0.5 mm; voxel size = 3 x 3 x 3.5 mm^3^; flip angle = 65°; field of view = 240 x 127.5 x 240 mm^2^; SENSE-factor = 2]. In addition, a field map and a high-resolution T1-weighted anatomical image was acquired.

fMRI data preprocessing and analysis were performed in the SPM12 toolbox. Preprocessing included distortion correction of functional images, slice time correction, and coregistration of the functional data to the T1-weighted image. The deformation fields derived from segmentation of the T1 image were used for normalization to the Montreal Neurological Institute (MNI)-152 template space. Last, smoothing with a 6 mm full-width-half-maximum kernel was applied to the functional data. Motion artifacts were assessed by calculating the framewise displacement (FD) values of each subject and task block (Power et al., 2012). Only subjects with FD < 0.5 mm were included in the analysis (mean 0.18 ± 0.04; no excluded participants based on this criterion). Moreover, single volumes with FD > 1 were censored in the statistical analyses using an additional binary regressor (mean 0.24 ± 0.35% of volumes excluded; and a maximum of 3.7% excluded in one participant).

### 2.6 Statistical analysis

#### 2.6.1 Task performance

The basic measures of accuracy (proportion of correct trials) and reaction times (RTs) for correct responses were averaged across thirds of 16 trials (one third of the total number of trials) for each block. Mixed-model analyses of variance (ANOVAs) were performed on these measures with third (1–3), block (1, 2), and task (A, B) as fixed effects and a random intercept by participant. Follow-up analyses examined the effects of the factor third in each task separately. Moreover, associations between task accuracy, RTs, model parameters, and cognitive tests were investigated with Pearson correlations and linear regressions. Spearman correlation values were used when data were not normally distributed.

#### 2.6.2 Model-based fMRI analysis

Two GLMs were conducted convolving stimuli and feedback onsets with the hemodynamic response function (as implemented in SPM12) and the different trial-based parameters from the model as parametric modulators. The parameters serving as parametric modulators were AS (used as modulator for stimuli onsets) and PE (used as modulator for feedback onsets). The onsets of stimuli and feedback in trials where no responses were given (“too late” responses) were added as an additional regressor of no interest. In addition, six realignment parameters from the data preprocessing were included as nuisance regressors and a binary regressor censored scans with FD > 1 (see MR Data Acquisition and Preprocessing). The fMRI analyses were conducted separately for each task because a direct comparison between them would be hardly interpretable, given differences in the visual load of stimuli and the levels of previous exposure (see Limitations).

For all whole-brain fMRI analyses, which examine voxels without a strong hypothesis, we applied a more stringent cluster-based family-wise error-corrected significance threshold of*p*_FWEc_= 0.05 with a cluster-defining threshold of*p*_CDT_= 0.001. The automated anatomical atlas (AAL) (Tzourio-Mazoyer et al., 2002) was used to provide anatomical labels to the MNI coordinates in these analyses.

The whole-brain analysis was further refined by a region of interest (ROI) analysis using FDR correction. ROIs were defined using the meta-analysis tool Neurosynth (Yarkoni et al., 2011) and are summarized inTable 1. The search terms “letter”, “audiovisual”, “learning”, and “error” were used to find the coordinates from areas related to reading, learning, and feedback processing areas in Neurosynth. When selecting the ROIs for analysis, we also considered the literature on the reading network (e.g.,Krishnan et al., 2016;Richlan, 2014) and the frontostriatal circuits of interest. This previous knowledge was used to constrain the number of ROIs and prevent us from performing a large number of statistical tests on smaller regions with less clear relevance to our study. In addition, we excluded areas that showed overlap between different keyword searches in Neurosynth. After downloading the images, we extracted the coordinates of the largest clusters identified in association tests provided by Neurosynth. These clusters indicate regions that have been consistently associated with our search terms in prior studies. Then we extracted the first eigenvariate of the time course of active voxels (*p*< 0.05) within a spherical search volume (*r*= 6 mm) around these coordinates. We used False Discovery Rate (FDR) to adjust*p*values in the ROI analysis of each task.

**Table 1.** Set of regions of interest based on Neurosynth online meta-analysis search tool.

	MNI coordinates		
Keyword/brain region	x	y	z
*“Letter”*			
L Fusiform gyrus	−44	−58	−14
R Fusiform gyrus ^a^	44	−58	−14
L Inferior frontal gyrus ^a^	−46	2	24
R Inferior frontal gyrus	46	2	24
*“Audiovisual”*			
L Superior temporal gyrus	−52	−22	8
R Superior temporal gyrus	54	−24	4
*“Learning” and “error”*			
L Putamen	−14	8	−10
R Putamen	26	2	−8
L Hippocampus	−22	−40	4
R Hippocampus	22	−32	6
L Caudate	−12	10	−10
R Caudate	14	10	−10
L Insula	−38	20	−6
R Insula	42	18	−6
L mid Cingulum	0	22	38
R mid Cingulum	4	26	40

a Contralateral regions were manually added and not found in the Neurosynth search.

L = left hemisphere; R = right hemisphere.

Additionally, the appendix presents a conventional fMRI analysis without using RLDDM parameters and dividing the onsets of stimuli and feedback in thirds of 16 trials.

## 3 Results

### 3.1 Behavioral data analyses: Learning performance

#### 3.1.1 Basic analysis of reaction time and accuracy

The main linear mixed models included the factors*task*(A,B),*third*(1,2,3), and*block*(1,2). They were followed by separate models examining learning and potential block effects in each task separately. The descriptive statistics of RT and accuracy (proportion of correct responses) are presented inTable 2andFigure 3.

**Table 2.** Task accuracy (proportion correct) and reaction times of correct responses.

	FBL-A	FBL-B
	*M (SD)*	*M (SD)*
Accuracy		
Third 1	0.61 (0.12)	0.57 (0.13)
Third 2	0.80 (0.16)	0.63 (0.18)
Third 3	0.85 (0.14)	0.77 (0.17)
Total	0.75 (0.17)	0.66 (0.18)
RTs (ms)		
Third 1	1365.08 (208.04)	1529.85 (228.42)
Third 2	1273.00 (198.13)	1553.02 (180.03)
Third 3	1256.97 (191.56)	1518.75 (207.85)
Total	1298.35 (204.16)	1533.87 (206.00)

**Fig. 3.**
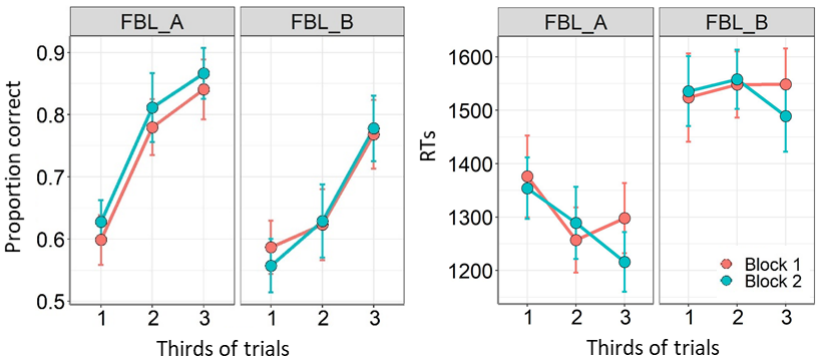
Accuracy (left plots) and RTs (right plots) averaged per trial thirds, separate lines per experimental block (red indicates first block; blue the second). Tasks A and B are presented in separate panels. Error bars represent 95% CIs.

##### 3.1.1.1 Accuracy

The analysis on the proportion of correct responses yielded a main effect of third (*F*(2,418) = 106.06,*p*< 0.001), indicating the increase in accuracy from the first to the last third of the blocks in both tasks. This learning curve is illustrated inFigure 3(left panel), seeTable 2for mean accuracy values. Additionally, there was a main effect of task (*F*(1,418) = 61.35,*p*< 0.001) and a significant interaction between task and third,*F*(1,418) = 9.37,*p*< 0.001, supporting a different learning curve between parts A and B, as shown inFigure 3. No other effects reached statistical significance in this model (*p’s*> 0.176). Further contrasts comparing the task in each third showed higher accuracy in FBL-A versus FBL-B in the third 2 (*t*(418) = 7.89,*p*< 0.001) and third 3 (*t*(418) = 3.74,*p*< 0.001); in third 1 this difference was only detected at trend levels (*p*= 0.055).

The differences in accuracies between FBL-A and FBL-B were further examined in separate models per task part. In both parts A and B, there was a significant main effect of third, (*F*(2,190) = 75.85,*p*< 0.001) and (*F*(2,190) = 43.58,*p*< 0.001), respectively. No block effects or interaction between thirds and blocks were found significant. The*t*pairwise comparisons between thirds in FBL-A showed significantly increased accuracy over both blocks from third 1 to third 2 (*t*(190) = 8.96,*p*< 0.001), and from third 2 to third 3 (*t*(190) = 2.85,*p*= 0.014). The pairwise comparisons on FBL-B data also showed significant increases from third 1 to third 2 (*t*(190) = 2.43,*p*= 0.042) and from third 2 to third 3 (*t*(190) = 6.59,*p*< 0.001).

**Fig. 4.**
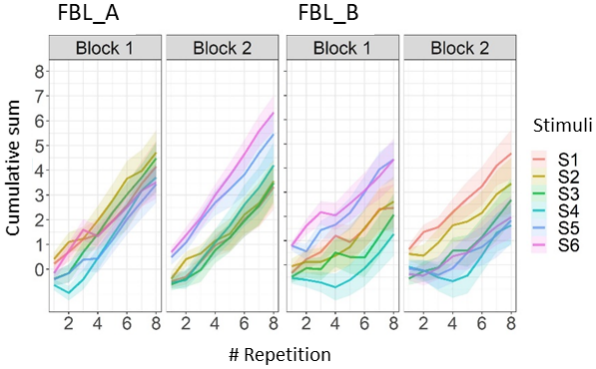
Learning trajectories per block and task for each unique sound. The values used in the calculation of the cumulative sum are +1 for trials with correct responses, -1 for incorrect trials, and 0 for trials with missing responses. Each sound is presented eight times. Shaded areas represent 95% CIs.

To further illustrate these learning curves for each stimulus pair,Figure 4shows the cumulative summed probabilities for each block and task.

##### 3.1.1.2 Reaction times

**Appendix Fig. A3.**
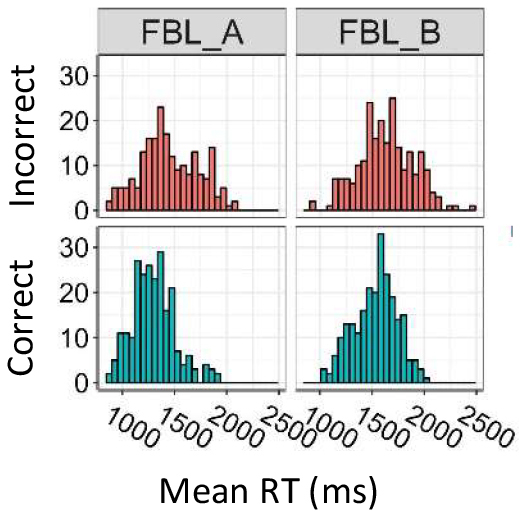
Histograms of reaction times over all blocks for correct (blue) and incorrect (red) responses. The left panel shows the data for task A and the right panel the data for task B.

The linear mixed model on the reaction times of correct responses revealed a main effect of third (*F*(2,418) = 6.46,*p*= 0.002) showing shorter RTs in the later trials of the blocks. The mean RTs are shown inTable 2andFigure 3(right panel). In addition, there was a main effect of task (*F*(1,418) = 300.32,*p*< 0.001) showing larger RTs in FBL-B than in FBL-A (see histograms inAppendix Fig. A3). The task effect was also found in interaction with third, (*F*(1,418) = 6.92,*p*= 0.001). In addition there was a significant interaction between third and block (*F*(2,418) = 4.03,*p*= 0.018), suggesting more pronounced effects of third in block 2 (follow-up pairwise comparison only showed significance in the third 2 versus third 3 comparison in block 2;*t*(417) = 2.74,*p*= 0.017). No other effects were significant in the main model,*p’s*> 0.179.

The separate analyses per task followed the main effect of task and the interaction between task and third. The analysis of FBL-A revealed a main effect of third (*F*(2,190) = 12.66,*p*< 0.001), suggesting shorter RTs as the block progressed (pairwise comparisons: third 1 vs. third 2,*t*(190) = -3.97,*p*< 0.001; third 2 vs. third 3 not significant,*p*= 0.769). There was also a trend for an interaction between third and block (*F*(2,190) = 3.03,*p*= 0.050), suggesting that this effect was more pronounced in block 2. The analysis on FBL-B revealed no significant effects or interactions on the mean RTs.

To sum up the basic performance analysis, we found FBL-B compared with FBL-A yielded longer RTs and lower accuracy. Both parts showed pronounced learning curves, that is, increased accuracy from first to last third of trials, but the increase was somewhat delayed in the FBL-B, and the accuracy differences between the tasks were mainly found in the second and last third.

#### 3.1.2 Reinforcement learning drift diffusion model

**Appendix Fig. A4.**
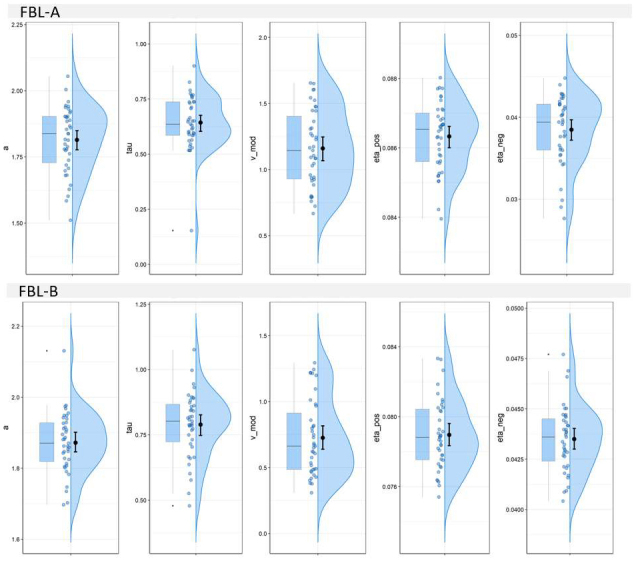
Distribution of the subject-level model parameters for tasks A and B. Error bars represent mean and 95% CI.

The model with dual learning showed a better fit for our data in the model comparisons (see Methods andAppendix Table A3). The posterior distributions of the subject-level parameters derived from the model are presented inFigure 5. The model parameter association strength for each pair of stimuli and the prediction error for correct trials are shown inFigure 6. Additional scatter plots and distribution of the model parameters are shown inAppendix Figure A4.

**Fig. 5.**
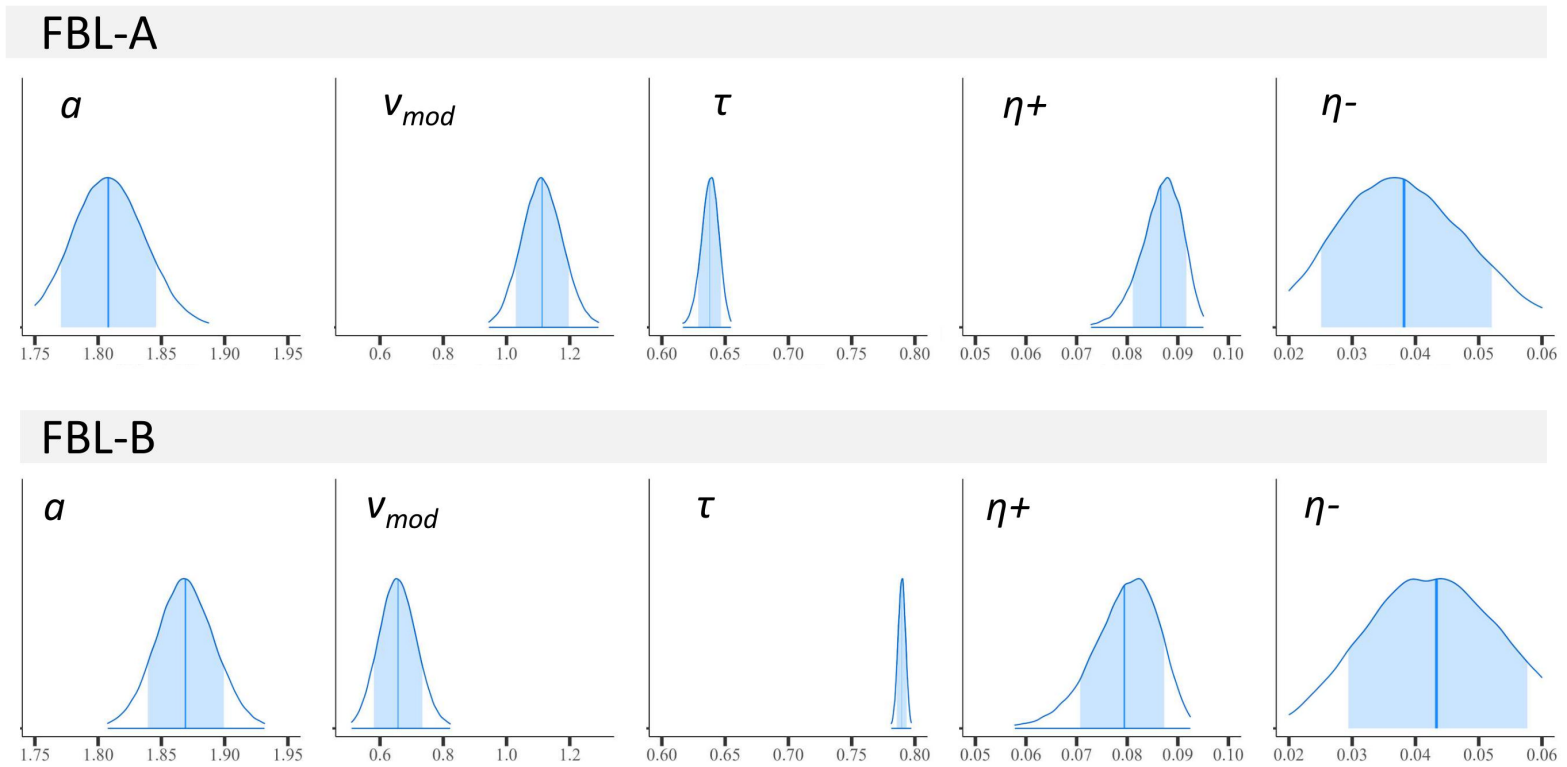
Posterior distributions of the subject-level parameters for each task. Shaded areas represent 80% credibility intervals, vertical line represents the mean point estimate.*a*= decision boundary;*v_mod_*= drift rate scaling factor;*τ*= nondecision time;*η*+ = learning rate for negative prediction errors;*η-*= learning rate for negative prediction errors.

**Fig. 6.**
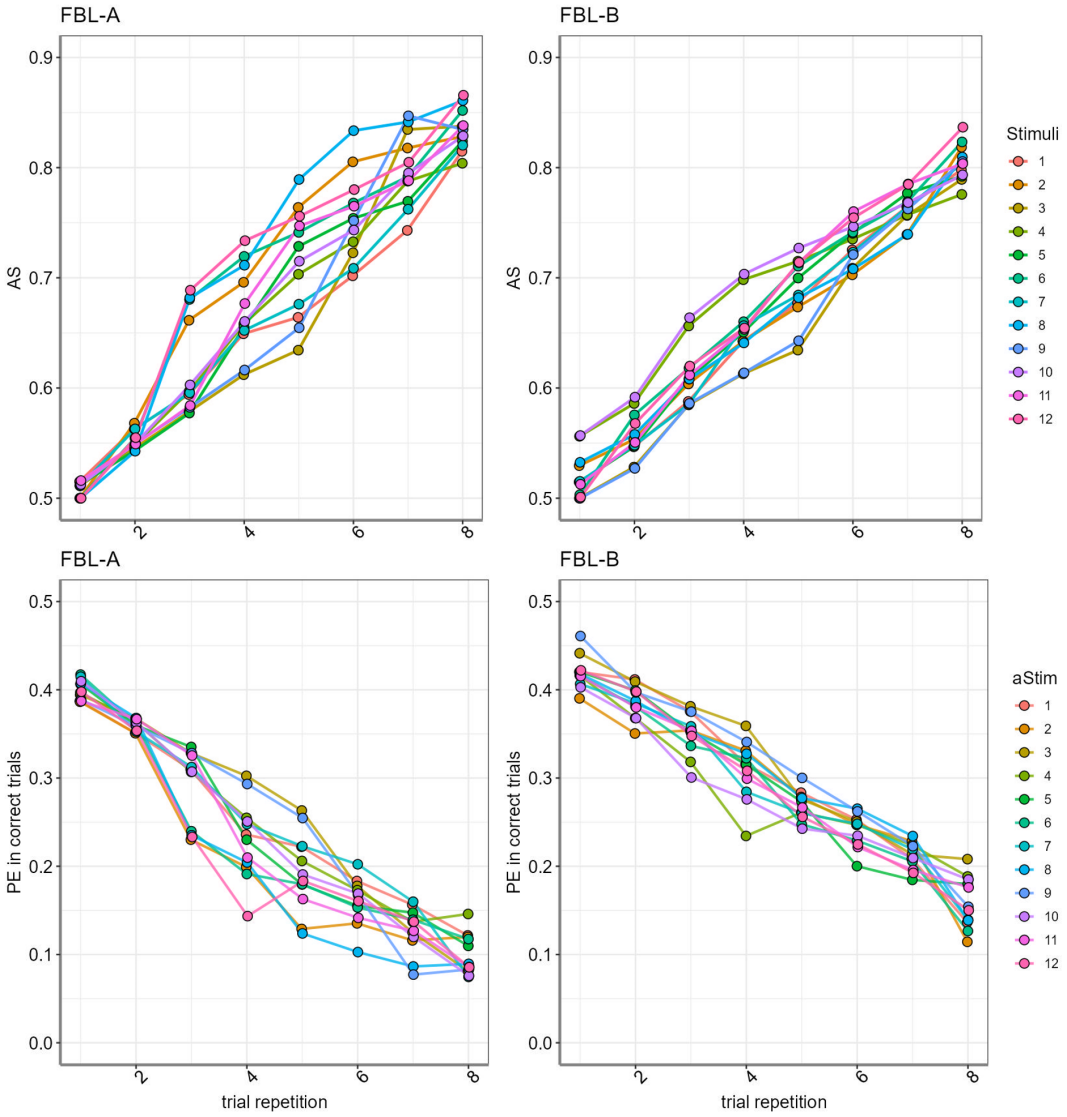
The top row shows the model parameter association strength (AS) for each possible pair of stimuli, increasing from 0 to 1 with each repetition of the pair. The bottom row shows the prediction error (PE) parameter for each possible pair of stimuli in the correct trials, the PE values approach zero with each repetition of the audiovisual pair. Tasks A and B are shown in the first and second column, respectively.

#### 3.1.3 Correlations between task performance and cognitive skills

As an additional analysis, we explored the correlations between performance in our experimental tasks (captured by RTs, accuracy, and model parameters) and cognitive abilities. This secondary analysis is reported in the Appendix (seeAppendix Table A4). There were potentially interesting associations indicating better task performance with higher nonverbal IQ, RAN, and text reading scores. However, the*p*values in those associations did not remain significant (under a*p*< 0.05 threshold) after applying an FDR correction for multiple testing (seeAppendix Table A4).

### 3.2 Changes in neural activations with learning

#### 3.2.1 Model-based fMRI

The goal of the main analysis in this study was to identify patterns of activation associated with trial-by-trial learning and prediction error. In the first-level analysis, the RLDDM parameters association strength (AS) and prediction error (PE) were mean centered and entered as parametric modulators of stimulus and feedback onsets, respectively, in the GLMs convolving the onsets with the hemodynamic response function.

##### 3.2.1.1 Model-based whole brain

Tables 3and4show suprathreshold clusters for adjusted*p_FWE_*< 0.05. The main activation clusters are also illustrated inFigure 7. The results show clusters in the visual cortex positively modulated by AS in both parts. A more extensive set of areas showed positive modulation by PE, including areas in the orbitofrontal cortex, striatal regions of putamen, caudate, and hippocampus as well as occipital and temporal regions, and a large cluster in the postcentral region. Both parts showed similar patterns although the extent of activations is more widespread in task A. Finally, there was a negative modulation by prediction error in the inferior frontal region, including the insula, and in task B in the supplementary motor area. The latter result was only detected in few voxels (k = 16) in task A (not reported in table).

**Table 3.** Results from model-based fMRI in FBL-A.

		MNI coordinates							
Contrast	Brain area	X	Y	Z	Cluster * p _FWE_*_cor_	Cluster k	Peak Z	Peak T	Peak * p _FWE_*_cor_
*Stimulus onset*									
AS+	Calcarine L	−11	−87	−3	<0.0001	112	6.46	8.81	<0.0001
	Lingual R	16	−72	−12	<0.0001	39	5.86	7.55	0.0002
*Feedback onset*									
PE+	Angular L	−50	−72	27	<0.0001	125	7.08	10.32	<0.0001
	Postcentral R	31	−33	48	<0.0001	1865	6.77	9.52	<0.0001
	Frontal Med Orb L	−8	42	−12	<0.0001	568	6.72	9.41	<0.0001
	Putamen L	−14	9	−9	<0.0001	60	6.54	8.98	<0.0001
	Sup Frontal L	−17	27	48	<0.0001	78	6.39	8.63	<0.0001
	Mid Frontal Orb L	−29	42	−12	<0.0001	59	6.31	8.46	<0.0001
	ParaHippocampal L	−17	−3	−27	<0.0001	67	6.12	8.06	<0.0001
	Precuneus R	7	−54	18	<0.0001	262	6.05	7.92	<0.0001
	Calcarine R	28	−63	18	<0.0001	44	5.83	7.48	0.0002
	Lingual L	−26	−45	3	<0.0001	35	5.7	7.23	0.0004
	Sup Temporal R	70	−3	6	<0.0001	58	5.66	7.16	0.0006
	Caudate R	19	3	24	<0.0001	59	5.51	6.89	0.0013
	Caudate L	−23	−3	21	<0.0001	30	5.42	6.72	0.0022
	ParaHippocampal R	25	−24	−21	<0.0001	30	5.38	6.65	0.0027
									
PE-	Inf Orbito Frontal R	37	24	−9	<0.0001	115	6.42	8.72	<0.0001

A voxel-wise threshold of*p_FWEcorr_*< 0.05 was used and only clusters with k > 30 are reported. Mid = middle; Sup = superior; Inf = inferior; Med = medial; Orb = orbital. AS/PE+ = positive modulation by association strength/prediction error; PE- = negative modulation by prediction error.

**Table 4.** Results from model-based fMRI in FBL-B.

		MNI coordinates							
Contrast	Brain area	X	Y	Z	Cluster * p _FWE_*_cor_	Cluster k	Peak Z	Peak T	Peak * p _FWE_*_cor_
*Stimulus onset*									
AS+	Mid Occipital L	−23	−90	3	<0.0001	164	6.23	8.3	<0.0001
	Calcarine R	22	−93	3	<0.0001	44	5.79	7.4	0.0003
*Feedback onset*									
PE+	Orbitofrontal R	19	15	−15	<0.0001	680	6.42	8.72	<0.0001
	Mid Occipital L	−44	−75	36	<0.0001	57	6.13	8.08	<0.0001
	Putamen L	−14	9	−12	<0.0001	51	6.11	8.04	<0.0001
	Hippocampus L	−35	−27	−12	<0.0001	79	6.09	7.99	<0.0001
	Caudate L	−14	27	−3	<0.0001	31	5.83	7.48	0.0002
	ParaHippocampal R	25	−9	−24	<0.0001	36	5.72	7.28	0.0004
	Frontal Med Orb R	1	51	−9	<0.0001	33	5.52	6.9	0.0012
PE-	Supp Motor Area R	4	6	63	<0.0001	113	6.55	9.01	<0.0001
	Inf Frontal R	49	21	3	<0.0001	98	6.23	8.3	<0.0001

A voxel-wise threshold of*p_FWEcorr_*< 0.05 was used and only clusters with k > 30 are reported. Mid = middle; Supp = supplementary; Inf = inferior; AS/PE+ = positive modulation by association strength/prediction error; PE- = negative modulation by prediction error.

**Fig. 7.**
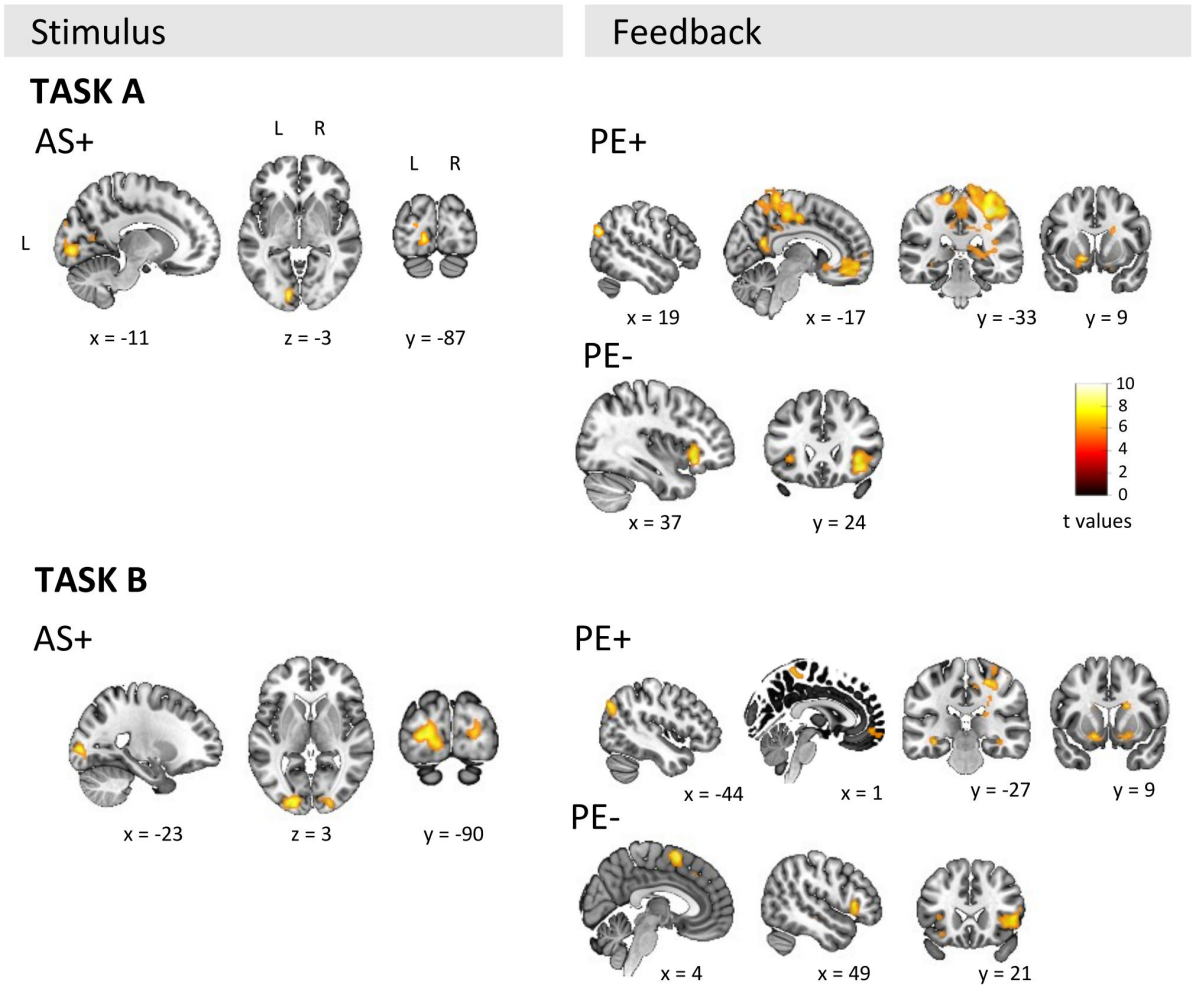
Suprathreshold clusters from the model-based analysis at*p_FWE_*_cor_< 0.05. Trial-level parameters from the computational model were used as parametric modulators. AS+ = positive modulation of associative strength in processing stimuli; PE+ = positive modulation of the prediction error in processing feedback; PE- = negative modulation of the prediction error in processing feedback; L = left; R = right. Coordinates in MNI-152 space.

##### 3.2.1.2 Model-based ROI

The ROI analysis is presented inTable 5andFigure 8. The results showed a positive modulation of activation during stimulus processing by AS in the left fusiform and left superior temporal gyrus in both parts. An additional effect in the left inferior frontal gyrus was found in task B. The results in the contralateral regions in the right hemisphere are shown inAppendix Figure A7. Regarding PE and feedback processing, there was positive modulation in bilateral putamen, caudate, and hippocampus in both tasks, and an additional effect in bilateral superior temporal gyrus was found in task A. In the opposite direction, insula and cingulum were negatively associated with PE.

**Table 5.** ROI*t*-tests against zero in model-based effects AS+ and PE+.

	FBL-A				FBL-B			
	Stimuli AS+		Feedback PE+		Stimuli AS+		Feedback PE+	
Keyword/ brain region	*t*	* p _FDR_*	*t*	* p _FDR_*	*t*	* p _FDR_*	*t*	* p _FDR_*
L Fusiform	**3.62**	**0.003**	1.44	0.231	**2.66**	**0.032**	0.53	0.788
R Fusiform	0.88	0.43	2.17	0.079	1.62	0.215	0.67	0.768
L IFG	1.89	0.117	0.79	0.464	**2.56**	**0.037**	−1.03	0.491
R IFG	1.74	0.144	−1.2	0.316	−0.27	0.866	−2.27	0.062
L STG	**3.04**	**0.011**	**2.43**	**0.046**	**2.58**	**0.037**	0.51	0.788
R STG	1.92	0.117	**4.27**	**<0.001**	**2.51**	**0.039**	1.88	0.136
L Putamen	1.95	0.117	**7.16**	**<0.001**	−0.01	0.995	**6.53**	**<0.001**
R Putamen	1.45	0.231	**4.45**	**<0.001**	1.18	0.424	**6.07**	**<0.001**
L Hippocampus	−1.08	0.34	**5.25**	**<0.001**	−0.38	0.866	**5.45**	**<0.001**
R Hippocampus	−0.34	0.733	**5.71**	**<0.001**	0.22	0.88	**4.98**	**<0.001**
L Caudate	1.17	0.316	**8.28**	**<0.001**	0.6	0.788	**9.29**	**<0.001**
R Caudate	1.79	0.138	**6.38**	**<0.001**	−1.16	0.424	**7.34**	**<0.001**
L Insula	1.3	0.280	**−4.76**	**<0.001**	−0.27	0.866	**−5.65**	**<0.001**
R Insula	1.15	0.316	**−6.83**	**<0.001**	0.17	0.891	**−5.82**	**<0.001**
L mid Cingulum	0.87	0.43	**−2.8**	**0.02**	0.51	0.788	**−4.14**	**<0.001**
R mid Cingulum	−0.68	0.519	**−3.97**	**<0.001**	0.33	0.866	**−5.67**	**<0.001**

Bold text indicates results with*p_FDR_*< 0.05 (False Discovery Rate adjustment for 32 tests).

mid Cingulum = medial cingulate gyrus; left hemisphere; R = right hemisphere; STG = superior temporal gyrus; IFG = inferior frontal gyrus; AS+ = positive modulation of association strength on stimuli processing; PE+ = positive modulation of prediction error on feedback processing.

**Fig. 8.**
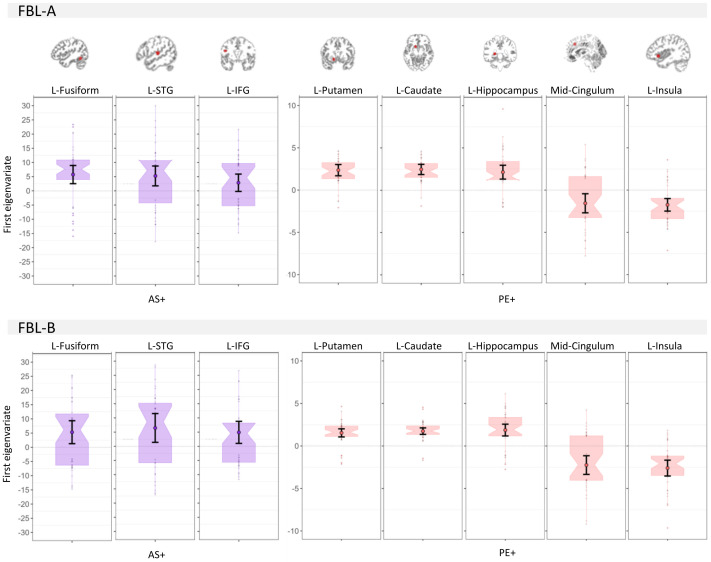
First eigenvariates for the region of interest (ROI) showing significant modulations by associative strength (AS) for processing stimuli and by prediction error (PE) on feedback presentations. Only left hemisphere ROIs are shown. L = left; STG = superior temporal gyrus; IFG = inferior frontal gyrus. Boxplots are notched around the median and error bars represent 95% CI. The embedded brain images show the corresponding ROI mask.

## 4 Discussion

The main goal of this study was to characterize the brain systems implicated in learning new symbol and speech sound associations and to assess individual differences in associative learning. For this, we used two tasks in which symbol–speech sound associations were learned via feedback on screen. As expected, we found that associations in task A were easier to learn than the ones of task B, in which diacritic marks were added to modify previously learned sounds. A reinforcement learning drift diffusion model was used to model performance in each task and the trial-by-trial parameters of association strength and prediction error were used as parametric modulators of stimulus and feedback processing, respectively, in the model-based fMRI analysis. The analysis yielded similar patterns of activations in both parts, showing how audiovisual associative learning contributes to visual specialization, the role of striatal regions in prediction error encoding, and the progressive engagement of cingulate and frontal regions related to performance monitoring.

### 4.1 Occipital responses to stimuli increase with strengthening of audiovisual associations

One of our goals was to examine specialization in visual/audiovisual regions when learning new associations within the short time scale of an experimental task. The model-based analysis revealed an occipital region that became more active for processing stimuli with increasing association strength. This region was located in the medial posterior portion of the occipital cortex with a peak in the left hemisphere (seeTables 3and4). The peak location approximates the letter-selective region selection described in a study using intracerebral recordings (Lochy et al., 2018). In that study, the electrode contacts that responded selectively to letters were widely distributed across regions of the ventral occipital cortex (e.g., inferior occipital, medial fusiform, middle temporal and inferior temporal gyrus, and other neighboring regions). The highest proportion of letter-selective intracerebral electrodes was located in the (left) posterior portion of the inferior occipital cortex, while a smaller, more anterior located group of electrodes detected selective responses in prelexical and lexical contrasts. A similar posterior-anterior gradient for letter–word selectivity was found in a previous fMRI study, which referred to a putative “letter-form area” in the posterior fusiform gyrus (Thesen et al., 2012). The peak of the cluster modulated by association strength in our model-based analysis has a more posterior and medial location similar to previous reports (Lochy et al., 2018;Thesen et al., 2012). Those studies also detected more anterior letter-selective regions, interpreted as letter form areas in their tasks, which included letter strings. Our ROI analysis also suggested that the activation extended to the left fusiform region.

Several factors may contribute to the posterior location in our results and the previously reported “letter form area”. The current study presents single characters instead of strings. In contrast to our relatively simple stimuli, more complex visual items could be expected to elicit stronger activations and possibly recruit a more extensive visual region. For example, a study comparing consonant–vowel–consonant strings to single letters yielded more activation in occipital regions to the string stimuli (Kronschnabel et al., 2014). Moreover, we used false fonts learned over a short experiment instead of real letters, and our task did not require semantic processing, as it was required inThesen et al. (2012)and which may explain the extension of their letter-selective response also to more anterior vOT areas. In relation to this, the left posterior middle temporal gyrus has been associated with lexical/semantic processing by previous work (Davey et al., 2016;Turken & Dronkers, 2011). In addition, a previous functional and structural MRI study found an area around the middle occipitotemporal sulcus that was sensitive to linguistic information, that is, contrasts involving real versus pseudowords and false fonts (Lerma-Usabiaga et al., 2018). Of note, the coordinates of the occipital region modulated by association strength in the current study were similar in both parts of the task, even though task B may require more focus on detailed features (diacritic marks). This would suggest that processing of visual features is not driving this result, although this would require further examination in a visual experiment with direct comparison between stimuli. Importantly, the modulation by the association strength parameter points at audiovisual learning, rather than just growing visual familiarity during the block, as a main contributor to the increase in visual activations.

To sum up, our model-based fMRI result supports results from developmental studies showing facilitated visual processing after audiovisual integration when learning how to read (Brem et al., 2010;Chyl et al., 2018;Dehaene-Lambertz et al., 2018;Fraga-González et al., 2021;Saygin et al., 2016). Also in line with this, but related to broader sensory learning and conditioning, top-down influences from auditory to visual cortex were found in a target detection task in which unknown to participants, auditory distractors predicted the presence or absence of visual stimuli (Den Ouden et al., 2009). We did not observe coactivation of audiovisual regions in the whole brain analysis with AS as parametric modulator, but the ROI analysis yielded activations of the superior temporal gyrus. Future studies could investigate whether the increased vOT activation is driven by input from auditory or audiovisual regions (such as STG) by analyzing functional connectivity between these areas. Along these lines, as suggested by the interactive account of the vOT (Price & Devlin, 2011), the observed vOT activations might be influenced by prediction error (PE) signals. However, our analyses did not find evidence of coactivation between vOT and frontostriatal PE regions during stimulus processing, although we did observe some effects during feedback onset (see below). It is possible that the short duration of our task, with relatively rapid learning, lacked sufficient power to detect these influences or to differentiate between distinct learning stages, such as those seen in reading acquisition, where initial learning and expert phases are more clearly defined.

**Appendix Fig. A5.**
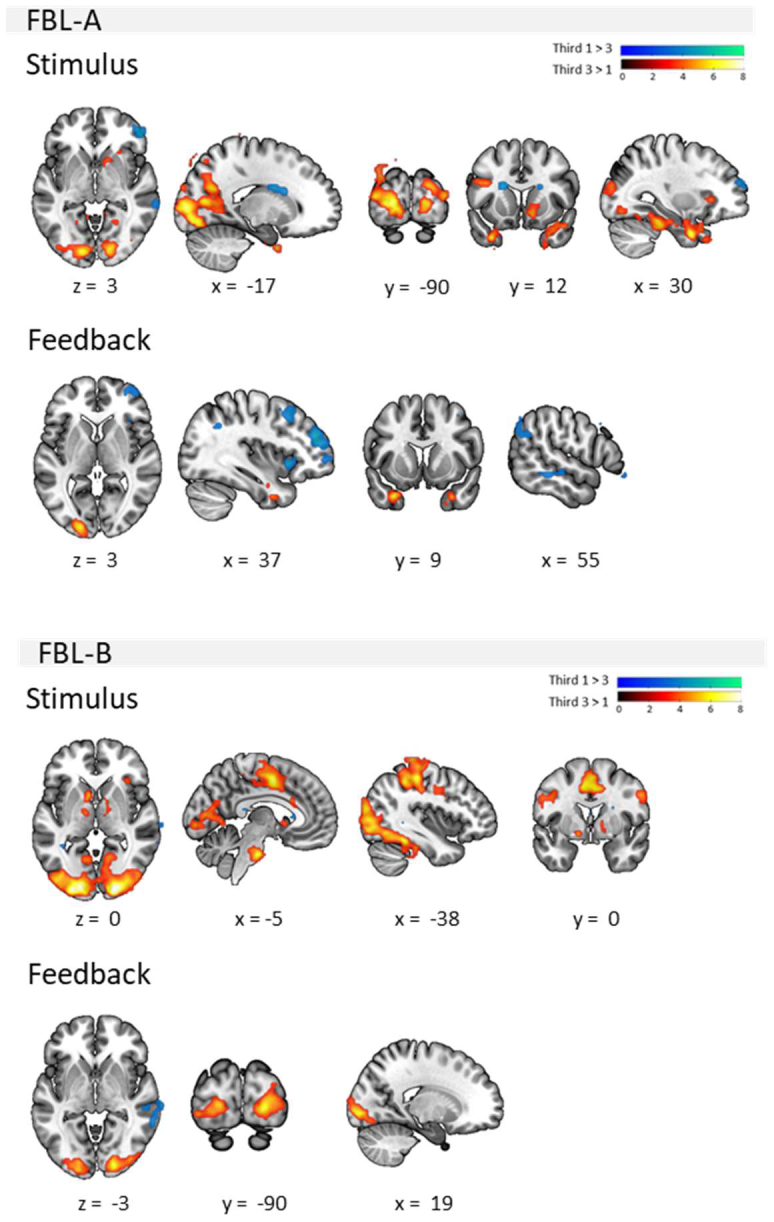
Suprathreshold activation clusters in FBL-A and FBL-B for stimulus and feedback processing in contrasts comparing the first versus last thirds of trials. Details are provided inAppendix Tables A5andA6*p_CDT_*< 0.001,*p_FWEc_*< 0.05. Coordinates in MNI-152 space.

**Appendix Fig. A7.**
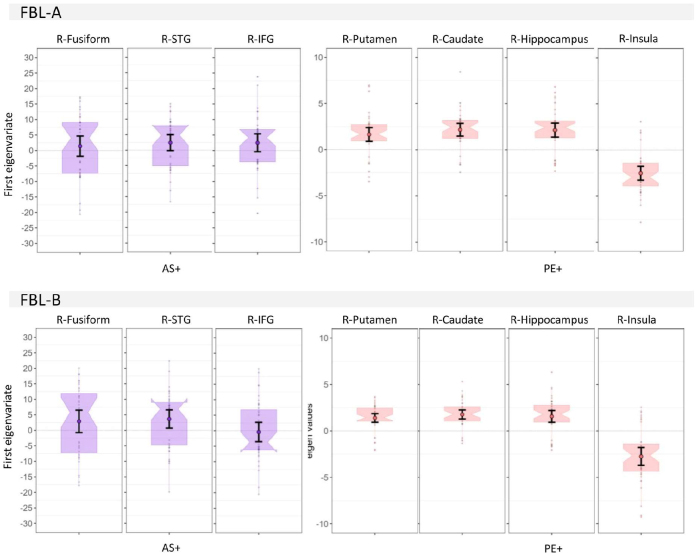
First eigenvariates for the region of interest (ROI) showing modulations by associative strength (AS) for processing stimuli and by prediction error (PE) on feedback presentations. Only right hemisphere ROIs are shown. R = right; STG = superior temporal gyrus; IFG = inferior frontal gyrus. Boxplots are notched around the median and error bars represent 95% CI.

Besides these findings, we should note that the supplementary, conventional analysis (seeAppendix Figs. A5toA6.aandA6.bandAppendix Tables A5toA6), using thirds of trials, confirmed stronger activations in extended areas of occipital and temporal regions in the consolidation phase compared with the initial trials of the block in both tasks. These effects may reflect enhanced representations after cross-modal learning, similar to those found with reading acquisition (Brem et al., 2010;Fraga-González et al., 2021;Hervais-Adelman et al., 2019;Taylor et al., 2019). We should note that from these analyses, we cannot rule out the influence of mere repeated exposure, although previous studies suggest repetition suppression effects rather than stronger activations to repeating, nondegraded visual stimuli (Müller et al., 2013). However, building up capacity to perceptually discriminate the symbols, that is, familiarity, could also be an alternative modulator of activations in the last part of the task (Xue et al., 2006). In addition, the whole-brain conventional analysis also showed inferior frontal and striatal regions, such as caudate and putamen, that were more active in the last part of the block. This suggests engagement of structures import for consolidating learning. Interestingly, the putamen was previously related to anticipation of outcomes from stimuli in a stimulus–action–reward learning task (Haruno & Kawato, 2006). Thus, these results may reflect stronger capacity to select the correct response as participants learnt the false font–speech sound associations.

### 4.2 Striatal regions involved in prediction error for audiovisual learning

Another goal of the current study was to investigate the learning mechanisms and brain regions contributing to visual/audiovisual specialization. Prediction error (PE) is proposed to drive learning by signaling the need to update predictions to minimize the discrepancy between expectations and a given outcome (Schultz & Dickinson, 2000). Thus, average PE decreases as predictions become more accurate and learning progresses (Friston, 2010). In the current setting, PE in each trial determines the change in association strength.

In both tasks, PE-dependent activations were found in several subcortical regions including putamen, caudate, and hippocampal region, as well as posterior occipital cortex and medial frontal cortex. In the ROI analysis, putamen, hippocampus, and caudate activity varied with the model-derived PE in both parts of the task. This finding supports the notion that striatal nuclei are involved in encoding PE signals (Garrison et al., 2013;Schultz, 2013). Besides sharing a common role in skill learning and memory (Hélie et al., 2015), the nuclei in the striatum have been associated with different connections and functions. For example, evidence suggests that caudate is functionally connected with frontal regions (Fjell et al., 2016;Grahn et al., 2008), and with orbitofrontal and cingulate cortices (Rieckmann et al., 2018). In contrast, the putamen has more basic sensorimotor connections (Fjell et al., 2016;Waldschmidt & Ashby, 2011). From the functional viewpoint, generally, anterior putamen, caudate, and their connections to association cortices are considered to be part of a broader cognitive system, while ventral striatal areas form a reward system and posterior putamen is more associated with a motor system (Rieckmann et al., 2018). It should be noted that the anatomical and functional boundaries between striatal systems are not always clear and, since PE is considered a rather ubiquitous mechanism that works at multiple levels, PE signals have been reported in many different regions depending on whether the focus is on motivational, attentional, cognitive, or perceptual responses (see review inDen Ouden et al., 2012). Close to the associative learning context of the current study,Den Ouden et al. (2009)found putamen and primary visual cortex to reflect the magnitude of surprise, that is, unsigned prediction errors, in their audiovisual task with conditioning.

The additional activations in the hippocampal region in the current study could also relate to the memory demands of the current task, especially in the learning phase when participants need to keep track of the different visual stimuli, the presented speech sound, as well as their response and subsequent feedback. Previous work suggested a role of hippocampus in selective attention (Mack et al., 2018) and in supporting working memory, especially when binding high resolution cross-modal information and handling complex features (Quak et al., 2015). Thus, the hippocampus would be relevant when considering the multisensory aspects of working memory (Quak et al., 2015) and the audiovisual nature of our task. In addition, an interaction between hippocampus and medial prefrontal cortex, both of which showed a positive association with PE in the current study, has also been suggested as key to memory formation and consolidation (Preston & Eichenbaum, 2013).

### 4.3 Prefrontal and cingulate regions negatively associated with prediction error

In both tasks, the ROIs insula and mid cingulum showed a negative association with PE (seeTable 4). The insula’s posterior regions connect to sensory regions and association cortex, while the anterior regions connect to anterior cingulate cortex, ventromedial prefrontal cortex, amygdala, and ventral striatum (Namkung et al., 2017). The anterior insula function has been associated with error awareness (Ullsperger et al., 2010), certainty and risk evaluation (Mohr et al., 2010), anticipation of outcomes (Preuschoff et al., 2008), and risk-averse behavior (Rudorf et al., 2012), as well as to integrate interoceptive signals with emotional, cognitive, and motivational signals (Namkung et al., 2017). Together with the anterior cingulate cortex, the anterior insula is proposed to be involved in evaluation of event/stimulus salience and oriented responses (see review inMenon & Uddin, 2010). The cingulate cortex, generally associated with conflict monitoring and feedback evaluation (Holroyd & Yeung, 2011), is proposed to facilitate response selection through its connections to mid-cingulate and supplementary motor area (Rudebeck et al., 2008). Additionally, the whole-brain analysis revealed that the cluster negatively influenced by PE included the adjacent supplementary motor area (suprathreshold voxels under FWE correction were detected in both tasks; however, the results for task A were not considered due to the small number of voxels (seeTables 3and4). Previous studies have linked this region to the learning of stimulus–response sequences, along with the striatum and middle frontal gyrus (Boettiger & D’Esposito, 2005).

### 4.4 Limitations

There are a few limitations worth mentioning in the current study design. The additional task B was incorporated in our paradigm to increase difficulty and simulate inconsistent symbol–speech sound associations with diacritic marks as speech sound modifiers. This resulted in the expected difference in performance, suggesting task B to be more demanding. However, a direct comparison of neural activations would be confounded by several factors. Each task presents differing visual loads: Task A involves recognizing single characters as false fonts, while task B requires identifying the main character and its corresponding modifier mark above. In addition, the main false fonts in B are not novel as they had been learned in the preceding block from part A. Our fMRI results from each task suggest similar activation and learning patterns; however, when considering the task requirements and brain activation results together, task A may offer a less ambiguous interpretative framework. Lastly, this study is constrained in its ability to address the secondary objectives and correlate task performance with pertinent reading skills. Despite variability in cognitive performance within the sample, most subjects performed within the normal skill range. Including individuals with poor reading skills, such as those with dyslexia or children with emergent reading skills would enable further assessment of the clinical potential of this paradigm, given the importance of early failure to establish letter–speech sound associations in reading disorders. Furthermore, our sample was recruited from the university and thus consisted of highly educated individuals. All participants except 5 had at least 3 additional years of education after the 11 years of compulsory education in Switzerland, 21 participants held a bachelor degree or higher. Thus, there was very limited heterogeneity in terms of participants’ education levels. Future studies would benefit from multicenter collaborations, allowing for the recruitment of significantly larger cohorts. This approach would address the challenge of sample heterogeneity and help overcome the broader issue of limited statistical power.

### 4.5 General conclusions

In conclusion, the current results directly link rapid specialization of visual areas to the learning of symbol–speech sound associations. This finding supports hypotheses from developmental and training studies that focus on broader time windows. In addition, we identified a set of striatal, cingulate, and prefrontal regions engaged in feedback and prediction error processing when learning novel symbol–speech sound associations. This represents a significant contribution to reading research, as studies addressing prediction errors in this specific context were largely absent. The current approach is well suited for further investigations involving clinical and developmental samples. In conclusion, we propose a framework to advance our understanding of the neurobiology involved in symbol/letter–speech sound binding, which is a crucial step when learning to read in alphabetic orthographies.

## Figures and Tables

**Appendix Fig. A6.a.**
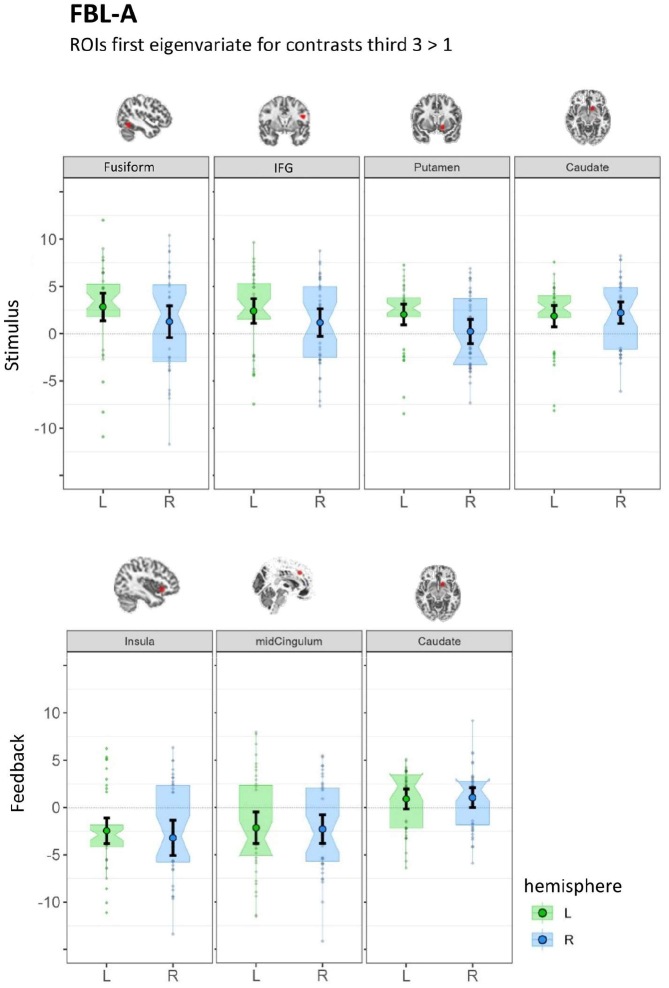
First eigenvariates for the region of interest (ROI) showing significant activations in the contrast third 3 > 1 for processing stimuli and feedback in FBL-A. Left hemisphere (L) regions are presented in green and their right (R) homologues in blue. Boxplots are notched around the median and error bars represent 95% CI. The embedded brain images show the corresponding ROI mask.

**Appendix Fig. A6.b.**
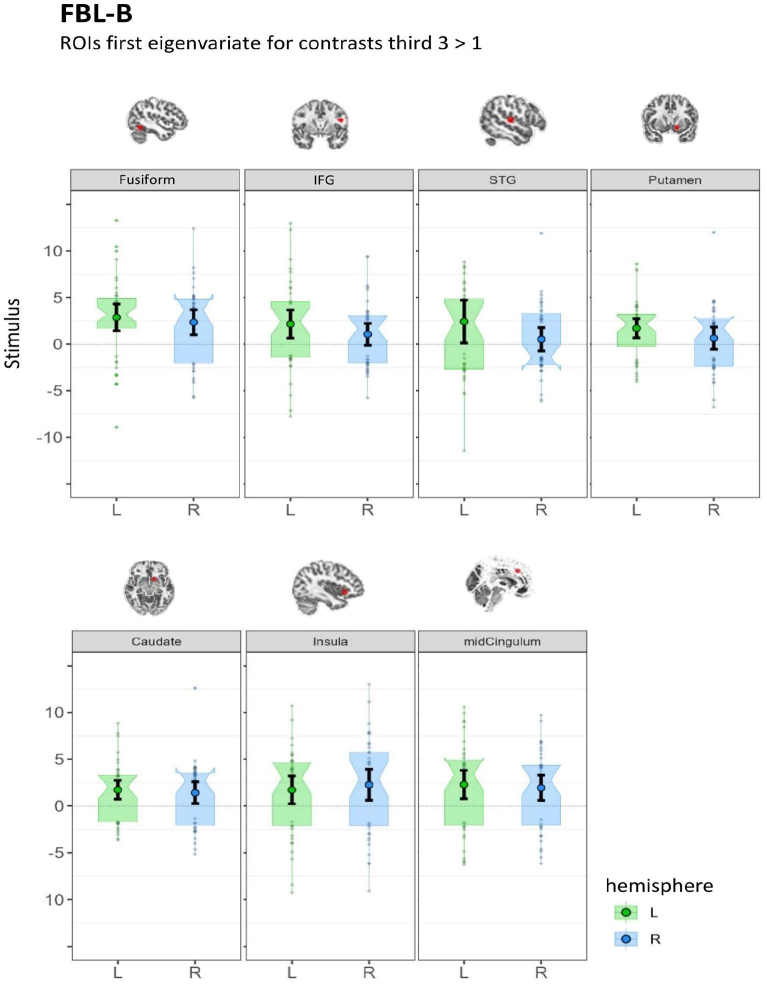
First eigenvariates for the region of interest (ROI) showing significant activations in the contrast third 3 > 1 for processing stimuli in FBL-B. Left hemisphere (L) regions are presented in green and their right (R) homologues in blue. Boxplots are notched around the median and error bars represent 95% CI. The embedded brain images show the corresponding ROI mask.

## Data Availability

The data and code for this publication are available onhttps://osf.io/kysgn/(DOI 10.17605/OSF.IO/KYSGN).

## Appendix

### A.1. Descriptive statistics

**Appendix Table A1.** Descriptive statistics showing cognitive performance.

	Group descriptives (N = 39)	
	*M* ( *SD* )	[min–max]
Age (years)	25.19 (3.12)	[18.14–32.51]
Female:male	21:18	
Nonverbal IQ (PR) ^a^	107.09 (6.09)	[96–119]
SLRT-II reading ^b^ (n correct items)		
Word ^b^	124.26 (13.9)	[102–161]
Pseudoword ^b^	84.5 (14.73)	[53–115]
SLRT-II (PR)		
Word	54.26 (25.49)	[13–99]
Pseudoword	65.47 (25.88)	[12–99]
Average	59.87 (24.16)	[22–99]
LGVT text reading (PR) ^b^		
Accuracy	71.05 (28.18)	[6–100]
Comprehension	55.45 (29.39)	[2–100]
Speed	50.13 (28.83)	[0–100]
RST spelling—raw	72.31 (5.79)	[52–80]
RST spelling—PR	84.1 (19.74)	[12–100]
Working memory—digit span ^b^		
Total span	12.59 (2.04)	[8–17]
Rapid automated naming (seconds) ^c^		
Colors	30.17 (5.69)	[20–48]
Objects	37.72 (5.99)	[26–53]

Final sample after exclusion (see criteria in Cognitive assessment):^a^N = 33;^b^N = 38;^c^N = 36.

PR = percentile score; SLRT-II = Salzburger Lese- und Rechtschreibtest II; LGVT = Lesegeschwindigkeits- und Verständnistest; RST = Rechtschreibetest- aktuelle Rechtschreiberegelung.

### A.2. RLDDM parameters

**Appendix Table A2.** Model parameters.

Parameter	Symbol	Value
Group-level priors		
Mean	$\mu_{d}$	~ *N* (0,1)
Standard deviation	$\sigma_{d}$	~ *N* (0,0.2)
Learning rates means	$\mu_{\eta +/-}$	~ *N* (0,0.3)
Learning rates std. deviation	$\sigma_{\eta +/-}$	~ *N* (0,0.5)
Subject-level priors		
Learning rates	$\eta_{+/-}$	~ *N* (0,1.5)
Decision boundary	*a*	~ * exp $(N(\mu_{a},\sigma_{a}$ * ))
Drift rate modulator	* ν _mod_*	~ * exp $(N(\mu_{v_{mod}},\sigma_{v_{mod}}$ * ))
Nondecision time	*τ*	~ | Φ $(N(\mu_{\tau },\sigma_{\tau }$ )) · $(RT_{min}-rb$ ) + rb |
Positive learning rate	* η _+_*	~ 0.1 · Φ $(N(\mu_{\eta +},\sigma_{\eta +}$ )
Negative learning rate	* η _-_*	~ 0.1 · Φ $(N(\mu_{\eta -},\sigma_{\eta -}$ )

### A.5. Table of model comparisons

**Appendix Table A3.** Model variation and comparison of fit estimates.

		FBL A			FBL B		
Model	η	*WAIC*	*-elpd*	*Rank*	*WAIC*	*-elpd*	*Rank*
RLDDM 1	Single	7017.29	3508.645	2	8491.748	4245.874	2
RLDDM 2	Dual	7003.93	3501.965	1	8484.534	4242.267	1

### A.7. Correlations between task performance and cognitive skills

Task performance was assessed by the basic measures (accuracy and reaction times averaged across blocks) and the subject-level parameters derived from the RLDDM (drift rate-*v_mod_*, decision boundary-*a,*nondecision time-*τ,*and learning rates η_+/-_). The main cognitive scores considered were nonverbal IQ, SLRT-II, LGTV speed, comprehension and accuracy, RST spelling, digit span, RAN colors, and objects (percentile scores were used when available). The uncorrected*p*values suggested moderate correlations between several cognitive tests (RAN, nonverbal IQ, and LGVT text reading comprehension and speed) and task performance, with Pearsons*R*ranging from 0.26 to 0.55. The statistical significance of these results, however, did not survive an FDR correction for multiple comparisons. They are presented inAppendix Table A4.

**Appendix Table A4.** Pearson correlations between task performance and cognitive skills with uncorrected*p*< 0.05.

Task performance	Cognitive test	*N*	*R*	*p*
**Basic measures**				
FBL A				
RT_total	Nonverbal IQ	32	−0.55	0.001
RT_total	RAN color (seconds)	34	0.42	0.012
Accuracy_total	Nonverbal IQ	33	0.49	0.004
Accuracy_total	LGVT comprehension PR	38	0.42	0.009
Accuracy_total	LGVT speed PR	38	0.40	0.013
Accuracy_total	RAN color (seconds)	35	−0.36	0.032
FBL B				
RT_total	RAN object	35	0.40	0.018
Accuracy_total	Nonverbal IQ	33	0.41	0.019
**RLDDM parameters**				
FBL A				
* v _mod_*	Nonverbal IQ	33	0.49	0.004
* v _mod_*	LGVT comprehension PR	38	0.36	0.025
* v _mod_*	LGVT speed PR	38	0.36	0.028
*a*	RAN color (seconds)	35	0.38	0.023
*a*	Nonverbal IQ	33	−0.38	0.029
FBL B				
* η _+_*	Nonverbal IQ	33	0.39	0.026
* v _mod_*	Nonverbal IQ	33	0.37	0.033

None of the*p*values remained significant after FDR correction.

FBL = feedback learning task; N = sample size after exclusion of outliers (1.5 IQR criterion); PR = percentile score; RT = reaction times;*v_mod_*= drift rate modulator;*a*= decision boundary;*τ*= nondecision time;*η*_+_= learning rate for correct responses; RAN = rapid automated naming; LGVT = Lesegeschwindigkeits- und Verständnistest (reading speed and comprehension test).

### A.8. Conventional fMRI analysis

A general linear model (GLM) using individual onsets of stimuli and feedback was convolved with the canonical hemodynamic response function as implemented in SPM12. The onsets of stimuli and feedback presentation were divided into thirds of 16 trials, which resulted in 6 regressors of interest in the GLM (thirds 1–3, stimuli/feedback onset; as a proxy to describe early learning vs. consolidated learning phases in the blocks). The onsets of stimuli and feedback in trials where no responses were given (“too late” responses) were added as an additional regressor of no interest. In addition, six realignment parameters from the data preprocessing were included as nuisance regressors and a binary regressor censored scans with FD > 1 (see MR Data Acquisition and Preprocessing). At the group level, the main effects of interest were the differences between third 3 and third 1 (defining*consolidation*vs.*learning*phases) in stimuli and feedback processing. This was tested with one-sample*t*tests using the respective contrast files of each subject. Labels of the resulting brain regions were obtained using the SPM anatomy toolbox^90^.

#### A.8.1. Whole brain

The whole-brain analysis yielded several patterns of activation associated with changes in stimulus and feedback processing along the experimental blocks. These results for each task are presented inAppendix Tables A5andA6andAppendix Figure A5.

##### A.8.1.1. FBL-A

Stimulus processing in the learning phase compared with the consolidation phase (third 1 > third 3) yielded significant activation clusters in right angular and temporal gyrus, as well as the dorsal portions of the caudate and midfrontal areas. In the consolidation phase (third 3 > third 1), stimulus processing resulted in extensive clusters of activations in bilateral occipital regions, including the occipitotemporal fusiform gyrus with a peak in the right fusiform. Additional clusters were found in right insula, right ventral portion of the caudate, and frontal areas. Feedback processing, however, resulted in stronger activations when comparing the learning versus consolidation phases (third 1 > third 3) in midfrontal areas, cerebellum, right insula, as well as in right midtemporal and angular gyri. In the consolidation phase (third 3 > third 1), clusters of activation were detected in left postcentral, right superior parietal, left midoccipital and temporal parietal cortex, right fusiform, and right superior orbitofrontal cortex.

**Appendix Table A5.** FBL-A.

	MNI coordinates					
Contrasts & brain areas	x	Y	z	Cluster * p _FWE_*_cor_	Voxels	Peak Z
*Stimulus onset*						
third 1 > third 3						
Dorsal Caudate R	22	6	21	0.007	83	5.14
Frontal Inf Orb R	43	48	−9	0.004	93	4.97
Angular R	61	−60	33	0.000	154	4.7
Dorsal Caudate L	−17	12	21	0.019	68	4.42
Mid Frontal R	37	48	30	0.015	72	4.3
Mid Temporal R	67	−24	−9	0.000	140	3.9
third 3 > third 1						
Sup Occipital L	−17	−90	3	0.000	3631	5.68
Fusiform R	31	3	−36	0.000	933	5.44
Insula R	37	−6	12	0.007	84	4.93
Supp Motor Area R	7	−6	54	0.000	339	4.56
Postcentral R	40	−36	72	0.000	247	4.52
Inferior Frontal L	−38	15	24	0.005	89	4.33
Ventral Caudate R	13	12	−9	0.007	85	4.23
*Feedback onset*						
third 1 > third 3						
Mid Frontal R	40	45	18	0.000	278	4.87
Cerebellum L	−41	−54	−45	0.009	75	4.79
Mid Frontal R	40	21	42	0.010	74	4.41
Insula R	37	18	−3	0.012	72	4.31
Angular R	43	−51	30	0.001	119	4.19
Mid Temporal R	55	−21	−9	0.001	107	3.9
third 3 > third 1						
Postcentral_L	−29	−30	75	0.000	390	5.18
Sup Parietal R	28	−57	75	0.007	79	5.15
Mid Occipital L	−20	−93	3	0.000	142	5.14
Mid Temporal Pole L	−29	9	−33	0.003	92	5.12
Sup Parietal L	−8	−84	60	0.013	70	4.63
Fusiform R	34	0	−36	0.030	58	4.53
Sup Orbito frontal R	16	48	−12	0.040	54	3.75

Significant activations at whole-brain cluster level with a cluster-defining threshold,*p*_CDT_< 0.001; FWEcor = Familywise error-corrected; mid Cingulum = medial cingulate gyrus; Sup = superior; Mid = middle; L = left hemisphere; R = right hemisphere.

##### A.8.1.2. FBL-B

Stimulus processing in the learning versus consolidation phase (third 1 > third 3) yielded no significant suprathreshold clusters. In the consolidation versus learning contrast (third 3 > third 1), there was a large activation cluster spanning occipital and occipitotemporal regions and a peak of activation at the right calcarine sulcus. Additionally, activation clusters covered the left and right postcentral gyri, right insula and pallidum and left mid Cingulum, caudate nucleus, superior parietal cortex, and amygdala.

Feedback processing in the learning phase (third 1 > third 3) only resulted in a cluster with a peak in the right midtemporal gyrus. In the consolidation phase (third 3 > third 1) activation peaks in the right calcarine and left lingual gyrus were detected.

**Appendix Table A6.** FBL-B.

	MNI coordinates					
Contrast & brain areas	X	y	z	Cluster * p _FWE_*_cor_	Voxels	Peak Z
*Stimulus onset*						
third 3 > third 1						
Calcarine R	22	−93	0	0.000	3569	6.58
Mid Cingulum L	−5	−3	48	0.000	687	5.61
Postcentral L	−38	−30	51	0.000	1070	5.37
Postcentral R	43	−21	42	0.000	556	5.26
Caudate L	−8	12	−3	0.009	79	4.97
Sup Parietal L	−11	−84	57	0.001	119	4.84
Insula R	34	24	6	0.015	70	4.81
Amygdala L	−14	0	−9	0.018	67	4.57
Pallidum R	13	0	3	0.010	77	3.99
*Feedback onset*						
third 1 > third 3						
Mid Temporal R	61	−27	−12	0.001	127	3.95
third 3 > third 1						
Calcarine R	19	−90	−3	0.000	340	5.28
Lingual L	−17	−90	0	0.000	279	4.76

Significant activations at whole-brain cluster level with a cluster-defining threshold,*p*_CDT_< 0.001; FWEcor = Familywise error-corrected; mid Cingulum = medial cingulate gyrus; Sup = superior; Mid = middle; L = left hemisphere; R = right hemisphere.

#### A.8.2. ROI

The contrast third 3 > 1 for stimulus and feedback onsets were examined in a set of ROIs defined by meta-analysis (see Statistical Analysis) to further specify the key regions showing changes in activation after learning in each task. The principal eigenvariate values of the con-images were extracted for each ROI and tested against zero using t-tests. The results are shown inAppendix Table A7andAppendix Figs. A6.aandA6.b. The results suggest increase in activation in third 3 compared with third 1 for processing stimuli in several reading network regions: in task A, this finding was confined to left fusiform and inferior frontal gyrus, but in task B, there was also involvement of right fusiform and left superior temporal gyrus. Regarding the areas involved in learning/performance monitoring, there was increased activation in caudate nuclei in both tasks, but in task B, there were significant effects in additional regions of insula and mid cingulate. For feedback processing, in contrast, there was decreased activation in the last third compared with the first third of the trials in right caudate, bilateral insula, and mid cingulum in FBL-A, but no significant effects were found in FBL-B.

**Appendix Table A7.** ROI t-tests in contrast third 3 > 1.

	FBL-A				FBL-B			
	Stimuli		Feedback		Stimuli		Feedback	
Keyword/brain region	*t*	*p*	*t*	*p*	*t*	*p*	*t*	*p*
L Fusiform	**3.95**	**<0.001**	0.24	0.81	**4.03**	**<0.001**	−0.13	0.898
R Fusiform	1.52	0.136	0.51	0.613	**3.51**	**0.001**	0.64	0.526
L IFG	**3.76**	**0.001**	0.24	0.811	**2.89**	**0.006**	0.58	0.567
R IFG	1.65	0.107	−1.82	0.076	1.84	0.074	−1.08	0.288
L STG	0.55	0.583	0.29	0.776	**2.13**	**0.039**	1.45	0.155
R STG	0.08	0.938	0.39	0.702	0.84	0.407	−0.37	0.713
L Putamen	**3.72**	**0.001**	2.03	0.049	**3.4**	**0.002**	−0.05	0.963
R Putamen	0.39	0.701	−0.77	0.449	1.08	0.289	−1.43	0.161
L Hippocampus	−1.39	0.173	0.74	0.464	−0.35	0.732	0.65	0.518
R Hippocampus	0.17	0.868	−0.16	0.877	−0.38	0.708	0.74	0.467
L Caudate	**3.34**	**0.002**	1.71	0.095	**3.42**	**0.002**	0.03	0.977
R Caudate	**3.93**	**<0.001**	**2.02**	**0.05**	**2.49**	**0.017**	0.01	0.991
L Insula	1.74	0.090	**−3.71**	**0.001**	**2.32**	**0.026**	−0.28	0.782
R Insula	−0.09	0.927	**−3.5**	**0.001**	**2.77**	**0.009**	−0.84	0.404
L mid Cingulum	0.25	0.802	**−2.63**	**0.012**	**3.03**	**0.004**	0.17	0.865
R mid Cingulum	−1.19	0.240	**−3.08**	**0.004**	**2.91**	**0.006**	−0.2	0.843

mid Cingulum = medial cingulate gyrus; left hemisphere; R = right hemisphere; STG = superior temporal gyrus; IFG = inferior frontal gyrus. Bold text indicates results with*pFDR*< 0.05 (False Discovery Rate adjustment for 32 tests).

## References

[b1] Alexander , W. H. , & Brown , J. W. ( 2019 ). The role of the anterior cingulate cortex in prediction error and signaling surprise . Topics in Cognitive Science , 11 ( 1 ), 119 – 135 . 10.1111/tops.12307 29131512

[b2] Altarelli , I. , Dehaene-Lambertz , G. , & Bavelier , D. ( 2020 ). Individual differences in the acquisition of non-linguistic audio-visual associations in 5 year olds . Developmental Science , 23 ( 4 ), e12913 . 10.1111/desc.12913 31608547

[b3] Boettiger , C. A. , & D’Esposito , M. ( 2005 ). Frontal networks for learning and executing arbitrary stimulus-response associations . Journal of Neuroscience , 25 ( 10 ), 2723 – 2732 . 10.1523/JNEUROSCI.3697-04.2005 15758182 PMC6725160

[b4] Bonte , M. , & Brem , S. ( 2024 ). Unraveling individual differences in learning potential: A dynamic framework for the case of reading development . Developmental Cognitive Neuroscience , 66 , 101362 . 10.1016/j.dcn.2024.101362 38447471 PMC10925938

[b5] Brem , S. , Bach , S. , Kucian , K. , Guttorm , T. K. , Martin , E. , Lyytinen , H. , Brandeis , D. , & Richardson , U. ( 2010 ). Brain sensitivity to print emerges when children learn letter-speech sound correspondences . Proceedings of the National Academy of Sciences of the United States of America , 107 ( 17 ), 7939 – 7944 . 10.1073/pnas.0904402107 20395549 PMC2867899

[b6] Brem , S. , Hunkeler , E. , Mächler , M. , Kronschnabel , J. , Karipidis , I. I. , Pleisch , G. , & Brandeis , D. ( 2018 ). Increasing expertise to a novel script modulates the visual N1 ERP in healthy adults . International Journal of Behavioral Development , 42 ( 3 ), 333 – 341 . 10.1177/0165025417727871

[b7] Brovelli , A. , Nazarian , B. , Meunier , M. , & Boussaoud , D. ( 2011 ). Differential roles of caudate nucleus and putamen during instrumental learning . NeuroImage , 57 ( 4 ), 1580 – 1590 . 10.1016/J.NEUROIMAGE.2011.05.059 21664278

[b8] Carpenter , B. , Gelman , A. , Hoffman , M. D. , Lee , D. , Goodrich , B. , Betancourt , M. , Brubaker , M. A. , Guo , J. , Li , P. , & Riddell , A. ( 2017 ). Stan: A probabilistic programming language . Journal of Statistical Software , 76 ( 1 ), 1 – 32 . 10.18637/jss.v076.i01 36568334 PMC9788645

[b9] Chyl , K. , Kossowski , B. , Dębska , A. , Łuniewska , M. , Banaszkiewicz , A. , Żelechowska , A. , Frost , S. J. , Mencl , W. E. , Wypych , M. , Marchewka , A. , Pugh , K. R. , & Jednoróg , K. ( 2018 ). Prereader to beginning reader: Changes induced by reading acquisition in print and speech brain networks . Journal of Child Psychology and Psychiatry, and Allied Disciplines , 59 ( 1 ), 76 – 87 . 10.1111/JCPP.12774 28691732 PMC5729096

[b10] Cignetti , F. , Nemmi , F. , Vaugoyeau , M. , Girard , N. , Albaret , J.-M. , Chaix , Y. , Péran , P. , & Assaiante , C. ( 2020 ). Intrinsic cortico-subcortical functional connectivity in developmental dyslexia and developmental coordination disorder . Cerebral Cortex Communications , 1 ( 1 ), 1 – 14 . 10.1093/texcom/tgaa011 PMC8152893 34296090

[b11] Davey , J. , Thompson , H. E. , Hallam , G. , Karapanagiotidis , T. , Murphy , C. , De Caso , I. , Krieger-Redwood , K. , Bernhardt , B. C. , Smallwood , J. , & Jefferies , E. ( 2016 ). Exploring the role of the posterior middle temporal gyrus in semantic cognition: Integration of anterior temporal lobe with executive processes . NeuroImage , 137 , 165 – 177 . 10.1016/j.neuroimage.2016.05.051 27236083 PMC4927261

[b12] Dehaene-Lambertz , G. , Monzalvo , K. , & Dehaene , S. ( 2018 ). The emergence of the visual word form: Longitudinal evolution of category-specific ventral visual areas during reading acquisition . PLoS Biology , 16 ( 3 ), e2004103 . 10.1371/journal.pbio.2004103 29509766 PMC5856411

[b13] Den Ouden , H. E. M. , Friston , K. , Daw , N. D. , McIntosh , A. R. , & Stephan , K. E. ( 2009 ). A dual role for prediction error in associative learning . Cerebral Cortex , 19 ( 5 ), 1175 – 1185 . 10.1093/cercor/bhn161 18820290 PMC2665159

[b14] Den Ouden , H. E. M. , Kok , P. , & de Lange , F. P . ( 2012 ). How prediction errors shape perception, attention, and motivation . Frontiers in Psychology , 3 ( DEC ), 548 . 10.3389/fpsyg.2012.00548 23248610 PMC3518876

[b16] Di Pietro , S. V. , Karipidis , I. I. , Pleisch , G. , & Brem , S. ( 2023 ). Neurodevelopmental trajectories of letter and speech sound processing from preschool to the end of elementary school . Developmental Cognitive Neuroscience , 61 , 101255 . 10.1016/j.dcn.2023.101255 37196374 PMC10203735

[b17] Fjell , A. M. , Sneve , M. H. , Storsve , A. B. , Grydeland , H. , Yendiki , A. , & Walhovd , K. B. ( 2016 ). Brain events underlying episodic memory changes in aging: A longitudinal investigation of structural and functional connectivity . Cerebral Cortex , 26 ( 3 ), 1272 – 1286 . 10.1093/cercor/bhv102 25994960 PMC4737610

[b18] Forstmann , B. U. , & Wagenmakers , E. J. ( 2015 ). Model-based cognitive neuroscience: A conceptual introduction . In B. Forstmann and E. J. Wagenmakers (Eds.), An introduction to model-based cognitive neuroscience (pp. 139 – 156 ). Springer . 10.1007/978-1-4939-2236-9_7

[b19] Fraga-González , G. , Pleisch , G. , Di Pietro , S. V. , Neuenschwander , J. , Walitza , S. , Brandeis , D. , Karipidis , I. I. , & Brem , S. ( 2021 ). The rise and fall of rapid occipito-temporal sensitivity to letters: Transient specialization through elementary school . Developmental Cognitive Neuroscience , 49 , 100958 . 10.1016/j.dcn.2021.100958 34010761 PMC8141525

[b20] Fraga-González , G. , Smit , D. J. A. , Molen , M. J. W. , Tijms , J. , Geus , E. J. C. , & Molen , M. W. ( 2019 ). Probability learning and feedback processing in dyslexia: A performance and heart rate analysis . Psychophysiology , 56 ( 12 ), e13460 . 10.1111/psyp.13460 31435961

[b21] Fraga-González , G. , Smit , D. J. A. , Van der Molen , M. J. W. , Tijms , J. , Stam , C. J. , de Geus , E. J. C. , & Van der Molen , M. W . ( 2022 ). Corrigendum: Graph analysis of EEG functional connectivity networks during a letter-speech sound binding task in adult dyslexics (Front. Psychol., (2021), 12, (767839), 10.3389/fpsyg.2021.767839) . Frontiers in Psychology , 12 , 5344 . 10.3389/fpsyg.2021.828043 PMC8658451 34899515

[b22] Fraga-González , G. , Žarić , G. , Tijms , J. , Bonte , M. , Blomert , L. , Leppänen , P. H. T. , & Van der Molen , M. W . ( 2016 ). Responsivity to dyslexia training indexed by the N170 amplitude of the brain potential elicited by word reading . Brain and Cognition , 106 , 42 – 54 . 10.1016/j.bandc.2016.05.001 27200495

[b23] Fraga-González , G. , Žarić , G. , Tijms , J. , Bonte , M. , & Van der Molen , M. W. ( 2017 ). Contributions of letter-speech sound learning and visual print tuning to reading improvement: Evidence from brain potential and dyslexia training studies . Brain Sciences , 7 ( 1 ), 10 . 10.3390/brainsci7010010 28106790 PMC5297299

[b24] Friston , K. J. ( 2010 ). The free-energy principle: A unified brain theory? Nature Reviews Neuroscience , 11 ( 2 ), 127 – 138 . 10.1038/nrn2787 20068583

[b25] Garrison , J. , Erdeniz , B. , & Done , J. ( 2013 ). Prediction error in reinforcement learning: A meta-analysis of neuroimaging studies . Neuroscience & Biobehavioral Reviews , 37 ( 7 ), 1297 – 1310 . 10.1016/J.NEUBIOREV.2013.03.023 23567522

[b26] Gelman , A. , Carlin , J. B. , Stern , H. S. , & Rubin , D. B. ( 1995 ). Bayesian data analysis . Chapman and Hall/CRC . 10.1201/9780429258411

[b27] Grahn , J. A. , Parkinson , J. A. , & Owen , A. M. ( 2008 ). The cognitive functions of the caudate nucleus . Progress in Neurobiology , 86 ( 3 ), 141 – 155 . 10.1016/J.PNEUROBIO.2008.09.004 18824075

[b28] Hancock , R. , Richlan , F. , & Hoeft , F. ( 2017 ). Possible roles for fronto-striatal circuits in reading disorder . Neuroscience and Biobehavioral Reviews , 72 , 243 – 260 . 10.1016/j.neubiorev.2016.10.025 27826071 PMC5189679

[b29] Haruno , M. , & Kawato , M. ( 2006 ). Different neural correlates of reward expectation and reward expectation error in the putamen and caudate nucleus during stimulus-action-reward association learning . Journal of Neurophysiology , 95 ( 2 ), 948 – 959 . 10.1152/JN.00382.2005/ASSET/IMAGES/LARGE/Z9K0010671730010.JPEG 16192338

[b30] Hashimoto , R. , & Sakai , K. L. ( 2004 ). Learning letters in adulthood: Direct visualization of cortical plasticity for forming a new link between orthography and phonology . Neuron , 42 ( 2 ), 311 – 322 . 10.1016/s0896-6273(04)00196-5 15091345

[b32] Hélie , S. , Ell , S. W. , & Ashby , F. G. ( 2015 ). Learning robust cortico-cortical associations with the basal ganglia: An integrative review . Cortex , 64 , 123 – 135 . 10.1016/j.cortex.2014.10.011 25461713

[b33] Hervais-Adelman , A. , Kumar , U. , Mishra , R. K. , Tripathi , V. N. , Guleria , A. , Singh , J. P. , Eisner , F. , & Huettig , F. ( 2019 ). Learning to read recycles visual cortical networks without destruction . Science Advances , 5 ( 9 ), 262 – 280 . 10.1126/sciadv.aax0262 PMC6750915 31555732

[b34] Holroyd , C. B. , & Verguts , T. ( 2021 ). The best laid plans: Computational principles of anterior cingulate cortex . Trends in Cognitive Sciences , 25 ( 4 ), 316 – 329 . 10.1016/J.TICS.2021.01.008 33593641

[b35] Holroyd , C. B. , & Yeung , N. ( 2011 ). An integrative theory of anterior cingulate cortex function: Option selection in hierarchical reinforcement learning . In R. B. Mars , J. Sallet , M. F. S. Rushworth , & N. Yeung (Eds.), Neural basis of motivational and cognitive control (pp. 332 – 349 ). The MIT Press . 10.7551/mitpress/9780262016438.003.0018

[b36] Horbach , J. , Scharke , W. , Cröll , J. , Heim , S. , & Günther , T. ( 2015 ). Kindergarteners’ performance in a sound-symbol paradigm predicts early reading . Journal of Experimental Child Psychology , 139 , 256 – 264 . 10.1016/j.jecp.2015.06.007 26166489

[b37] Ibrahimović , N. , & Bulheller , S. ( 2013 ). Rechtschreibtest RST-ARR: Aktuelle Rechtschreibregelung: Lückendiktate. Pearson Assessment & Information. https://www.pearsonclinical.de/rst.html

[b38] Karipidis , I. I. , Pleisch , G. , Brandeis , D. , Roth , A. , Röthlisberger , M. , Schneebeli , M. , Walitza , S. , & Brem , S. ( 2018 ). Simulating reading acquisition: The link between reading outcome and multimodal brain signatures of letter–speech sound learning in prereaders . Scientific Reports , 8 ( 1 ), 7121 . 10.1038/s41598-018-24909-8 29740067 PMC5940897

[b39] Krishnan , S. , Watkins , K. E. , & Bishop , D. V. M. ( 2016 ). Neurobiological basis of language learning difficulties . Trends in Cognitive Sciences , 20 ( 9 ), 701 – 714 . 10.1016/j.tics.2016.06.012 27422443 PMC4993149

[b40] Kronschnabel , J. , Brem , S. , Maurer , U. , & Brandeis , D. ( 2014 ). The level of audiovisual print-speech integration deficits in dyslexia . Neuropsychologia , 62 , 245 – 261 . 10.1016/j.neuropsychologia.2014.07.024 25084224

[b41] Lefly , D. L. , & Pennington , B. F. ( 2000 ). Reliability and validity of the adult reading history questionnaire . Journal of Learning Disabilities , 33 ( 3 ), 286 – 296 . 10.1177/002221940003300306 15505966

[b42] Lerma-Usabiaga , G. , Carreiras , M. , & Paz-Alonso , P. M. ( 2018 ). Converging evidence for functional and structural segregation within the left ventral occipitotemporal cortex in reading . Proceedings of the National Academy of Sciences of the United States of America , 201803003. 10.1073/pnas.1803003115 PMC6196482 30224475

[b43] Liljeholm , M. , & O’Doherty , J. P. ( 2012 ). Contributions of the striatum to learning, motivation, and performance: An associative account . Trends in Cognitive Sciences , 16 ( 9 ), 467 – 475 . 10.1016/J.TICS.2012.07.007 22890090 PMC3449003

[b44] Lochy , A. , Jacques , C. , Maillard , L. , Colnat-Coulbois , S. , Rossion , B. , & Jonas , J. ( 2018 ). Selective visual representation of letters and words in the left ventral occipito-temporal cortex with intracerebral recordings . Proceedings of the National Academy of Sciences of the United States of America , 115 ( 32 ), E7595 – E7604 . 10.1073/pnas.1718987115 30038000 PMC6094145

[b45] Luce , R. ( 1959 ). Individual choice behavior: A theoretical analysis . John Willey and Sons . 10.1037/14396-000

[b46] Machina , M. J. ( 1987 ). Choice under uncertainty: Problems solved and unsolved . Journal of Economic Perspectives , 1 ( 1 ), 121 – 154 . 10.1257/JEP.1.1.121

[b47] Mack , M. L. , Love , B. C. , & Preston , A. R. ( 2018 ). Building concepts one episode at a time: The hippocampus and concept formation . Neuroscience Letters , 680 , 31 – 38 . 10.1016/J.NEULET.2017.07.061 28801273 PMC5803467

[b48] Maurer , U. , Brem , S. , Bucher , K. , & Brandeis , D. ( 2005 ). Emerging neurophysiological specialization for letter strings . Journal of Cognitive Neuroscience , 17 ( 10 ), 1532 – 1552 . 10.1162/089892905774597218 16269095

[b49] Mayer , A. ( 2011 ). Test zur Erfassung der phonologischen Bewusstheit und der Benenngeschwindigkeit (TEPHOBE) . Ernst Reinhardt Verlag. ISBN: 978-3-497-03225-9

[b50] Menon , V. , & Uddin , L. Q. ( 2010 ). Saliency, switching, attention and control: A network model of insula function . Brain Structure & Function , 214 ( 5–6 ), 655 – 667 . 10.1007/S00429-010-0262-0 20512370 PMC2899886

[b51] Mohr , P. N. C. , Biele , G. , & Heekeren , H. R. ( 2010 ). Neural processing of risk . Journal of Neuroscience , 30 ( 19 ), 6613 – 6619 . 10.1523/JNEUROSCI.0003-10.2010 20463224 PMC6632558

[b52] Moll , K. , & Landerl , K. ( 2010 ). SLRT-II: Lese-und Rechtschreibtest . Bern : Verlag Hans Huber .

[b54] Müller , N. G. , Strumpf , H. , Scholz , M. , Baier , B. , & Melloni , L. ( 2013 ). Repetition suppression versus enhancement—It’s quantity that matters . Cerebral Cortex , 23 ( 2 ), 315 – 322 . 10.1093/CERCOR/BHS009 22314047

[b55] Namkung , H. , Kim , S. H. , & Sawa , A. ( 2017 ). The insula: An underestimated brain area in clinical neuroscience, psychiatry, and neurology . Trends in Neurosciences , 40 ( 4 ), 200 – 207 . 10.1016/j.tins.2017.02.002 28314446 PMC5538352

[b56] Nicolson , R. I. , & Fawcett , A. J. ( 2007 ). Procedural learning difficulties: Reuniting the developmental disorders? Trends in Neurosciences , 30 ( 4 ), 135 – 141 . 10.1016/j.tins.2007.02.003 17328970

[b57] Norton , E. S. , Beach , S. D. , & Gabrieli , J. D. ( 2015 ). Neurobiology of dyslexia . Current Opinion in Neurobiology , 30 , 73 – 78 . 10.1016/j.conb.2014.09.007 25290881 PMC4293303

[b58] Pasqualotto , A. , Cochrane , A. , Bavelier , D. , & Altarelli , I. ( 2021 ). A novel non-linguistic audio-visual learning paradigm to test the cognitive correlates of learning rate . Proceedings of the Annual Meeting of the Cognitive Science Society , 43 . https://escholarship.org/uc/item/5pj9t90k

[b59] Pedersen , M. L. , & Frank , M. J. ( 2020 ). Simultaneous hierarchical bayesian parameter estimation for reinforcement learning and drift diffusion models: A tutorial and links to neural data . Computational Brain and Behavior , 3 ( 4 ), 458 – 471 . 10.1007/s42113-020-00084-w 35128308 PMC8811713

[b60] Pedersen , M. L. , Frank , M. J. , & Biele , G. ( 2017 ). The drift diffusion model as the choice rule in reinforcement learning . Psychonomic Bulletin and Review , 24 ( 4 ), 1234 – 1251 . 10.3758/S13423-016-1199-Y 27966103 PMC5487295

[b61] Perrone-Bertolotti , M. , Vidal , J. R. , de Palma , L. , Hamamé , C. M. , Ossandon , T. , Kahane , P. , Minotti , L. , Bertrand , O. , & Lachaux , J. P. ( 2014 ). Turning visual shapes into sounds: Early stages of reading acquisition revealed in the ventral occipitotemporal cortex . NeuroImage , 90 , 298 – 307 . 10.1016/j.neuroimage.2013.12.027 24370818

[b62] Pleisch , G. , Karipidis , I. I. , Brauchli , C. , Röthlisberger , M. , Hofstetter , C. , Stämpfli , P. , Walitza , S. , & Brem , S. ( 2019 ). Emerging neural specialization of the ventral occipitotemporal cortex to characters through phonological association learning in preschool children . NeuroImage , 189 , 813 – 831 . 10.1016/j.neuroimage.2019.01.046 30677503

[b63] Power , J. D. , Barnes , K. A. , Snyder , A. Z. , Schlaggar , B. L. , & Petersen , S. E. ( 2012 ). Spurious but systematic correlations in functional connectivity MRI networks arise from subject motion . NeuroImage , 59 ( 3 ), 2142 – 2154 . 10.1016/J.NEUROIMAGE.2011.10.018 22019881 PMC3254728

[b64] Preston , A. R. , & Eichenbaum , H. ( 2013 ). Interplay of hippocampus and prefrontal cortex in memory . Current Biology , 23 ( 17 ), R764 – R773 . 10.1016/J.CUB.2013.05.041 24028960 PMC3789138

[b65] Preuschoff , K. , Quartz , S. R. , & Bossaerts , P. ( 2008 ). Human insula activation reflects risk prediction errors as well as risk . Journal of Neuroscience , 28 ( 11 ), 2745 – 2752 . 10.1523/JNEUROSCI.4286-07.2008 18337404 PMC6670675

[b111] Price , C. J. , & Devlin , J. T. ( 2011 ). The interactive account of ventral occipitotemporal contributions to reading . Trends in Cognitive Sciences , 15 ( 6 ), 246 – 253 . 10.1016/j.tics.2011.04.001 21549634 PMC3223525

[b66] Quak , M. , London , R. E. , & Talsma , D. ( 2015 ). A multisensory perspective of working memory . Frontiers in Human Neuroscience , 9 ( APR ), 1 – 11 . 25954176 10.3389/fnhum.2015.00197 PMC4404829

[b67] Ratcliff , R. ( 1978 ). A theory of memory retrieval . Psychological Review , 85 ( 2 ), 59 – 108 . 10.1037/0033-295X.85.2.59

[b68] Ratcliff , R. , Smith , P. L. , Brown , S. D. , & McKoon , G. ( 2016 ). Diffusion decision model: Current issues and history . Trends in Cognitive Sciences , 20 ( 4 ), 260 – 281 . 10.1016/J.TICS.2016.01.007 26952739 PMC4928591

[b69] Rescorla , R. A. , & Wagner , A. R. ( 1972 ). A Theory of Pavlovian conditioning: Variations in the effectiveness of reinforcement and nonreinforcement . In A. H. Black , & W. F. Prokasy (Eds.), Classical conditioning II: Current research and theory (pp. 64 – 99 ). New York : Appleton-Century-Crofts .

[b70] Reynolds , C. R. , & Kamphaus , R. W. ( 2003 ). Reynolds Intellectual Assessment Scales: Professional manual . Psychological Assessment Resources . 10.1016/j.acn.2003.10.001

[b71] Richlan , F. ( 2014 ). Functional neuroanatomy of developmental dyslexia: The role of orthographic depth . Frontiers in Human Neuroscience , 8 ( May ), 347 . 10.3389/fnhum.2014.00347 24904383 PMC4033006

[b72] Richlan , F. ( 2019 ). The functional neuroanatomy of letter-speech sound integration and its relation to brain abnormalities in developmental dyslexia . Frontiers in Human Neuroscience , 13 , 21 . 10.3389/fnhum.2019.00021 30774591 PMC6367238

[b73] Rieckmann , A. , Johnson , K. A. , Sperling , R. A. , Buckner , R. L. , & Hedden , T. ( 2018 ). Dedifferentiation of caudate functional connectivity and striatal dopamine transporter density predict memory change in normal aging . Proceedings of the National Academy of Sciences of the United States of America , 115 ( 40 ), 10160 – 10165 . 10.1073/pnas.1804641115 30224467 PMC6176586

[b74] Rudebeck , P. H. , Behrens , T. E. , Kennerley , S. W. , Baxter , M. G. , Buckley , M. J. , Walton , M. E. , & Rushworth , M. F. S. ( 2008 ). Frontal cortex subregions play distinct roles in choices between actions and stimuli . Journal of Neuroscience , 28 ( 51 ), 13775 – 13785 . 10.1523/JNEUROSCI.3541-08.2008 19091968 PMC6671924

[b75] Rudorf , S. , Preuschoff , K. , & Weber , B. ( 2012 ). Neural correlates of anticipation risk reflect risk preferences . Journal of Neuroscience , 32 ( 47 ), 16683 – 16692 . 10.1523/JNEUROSCI.4235-11.2012 23175822 PMC6621765

[b76] Saygin , Z. M. , Osher , D. E. , Norton , E. S. , Youssoufian , D. A. , Beach , S. D. , Feather , J. , Gaab , N. , Gabrieli , J. D. , & Kanwisher , N. ( 2016 ). Connectivity precedes function in the development of the visual word form area . Nature Neuroscience , 19 ( 9 ), 1250 – 1255 . 10.1038/nn.4354 27500407 PMC5003691

[b77] Schneider , W. , Schlagmuller , M. , & Ennemoser , M. ( 2017 ). Lesegeschwindigkeits-und Verständnistest für die Klassen 5 -12+ (LGVT 5-12+) [Reading speed and comprehension test for grades 5-12+] . Hogrefe . https://econtent.hogrefe.com/do/10.5555/t0148801/abs/

[b78] Schultz , W. ( 2013 ). Updating dopamine reward signals . Current Opinion in Neurobiology , 23 ( 2 ), 229 – 238 . 10.1016/J.CONB.2012.11.012 23267662 PMC3866681

[b79] Schultz , W. , & Dickinson , A. ( 2000 ). Neuronal coding of prediction errors . Annual Review of Neuroscience , 23 , 473 – 500 . 10.1146/annurev.neuro.23.1.473 10845072

[b80] Spratling , M. W. ( 2017 ). A review of predictive coding algorithms . Brain and Cognition , 112 , 92 – 97 . 10.1016/j.bandc.2015.11.003 26809759

[b81] Stan Development Team . ( 2021 ). CmdStan: The command-line interface to Stan . https://mc-stan.org/

[b82] Taylor , J. S. H. , Davis , M. H. , & Rastle , K. ( 2019 ). Mapping visual symbols onto spoken language along the ventral visual stream . Proceedings of the National Academy of Sciences of the United States of America , 3 ( 36 ), 17723 – 17728 . 10.1073/pnas.1818575116 PMC6731646 31427523

[b83] Thesen , T. , McDonald , C. R. , Carlson , C. , Doyle , W. , Cash , S. , Sherfey , J. , Felsovalyi , O. , Girard , H. , Barr , W. , Devinsky , O. , Kuzniecky , R. , & Halgren , E. ( 2012 ). Sequential then interactive processing of letters and words in the left fusiform gyrus . Nature Communications , 3 , 1284 . 10.1038/ncomms2220 PMC4407686 23250414

[b84] Turken , A. U. , & Dronkers , N. F. ( 2011 ). The neural architecture of the language comprehension network: Converging evidence from lesion and connectivity analyses . Frontiers in Systems Neuroscience , 5 , 1 . 10.3389/fnsys.2011.00001 21347218 PMC3039157

[b85] Tzourio-Mazoyer , N. , Landeau , B. , Papathanassiou , D. , Crivello , F. , Etard , O. , Delcroix , N. , Mazoyer , B. , & Joliot , M. ( 2002 ). Automated anatomical labeling of activations in SPM using a macroscopic anatomical parcellation of the MNI MRI single-subject brain . NeuroImage , 15 ( 1 ), 273 – 289 . 10.1006/nimg.2001.0978 11771995

[b86] Ullsperger , M. , Harsay , H. A. , Wessel , J. R. , & Ridderinkhof , K. R. ( 2010 ). Conscious perception of errors and its relation to the anterior insula . Brain Structure and Function , 214 ( 5–6 ), 629 – 643 . 10.1007/s00429-010-0261-1 20512371 PMC2886909

[b87] van Atteveldt , N. , & Ansari , D. ( 2014 ). How symbols transform brain function: A review in memory of Leo Blomert . Trends in Neuroscience and Education , 3 ( 2 ), 44 – 49 . 10.1016/J.TINE.2014.04.001

[b88] Vandekerckhove , J. , Tuerlinckx , F. , & Lee , M. D. ( 2011 ). Hierarchical diffusion models for two-choice response times . Psychological Methods , 16 ( 1 ), 44 – 62 . 10.1037/A0021765 21299302

[b90] Waldschmidt , J. G. , & Ashby , F. G. ( 2011 ). Cortical and striatal contributions to automaticity in information-integration categorization . NeuroImage , 56 ( 3 ), 1791 – 1802 . 10.1016/J.NEUROIMAGE.2011.02.011 21316475 PMC3085658

[b91] Wang , F. , Karipidis , I. I. , Pleisch , G. , Fraga-González , G. , & Brem , S. ( 2020 ). Development of print-speech integration in the brain of beginning readers with varying reading skills . Frontiers in Human Neuroscience , 14 , 289 . 10.3389/fnhum.2020.00289 32922271 PMC7457077

[b92] Watanabe , S. ( 2010 ). Asymptotic equivalence of Bayes cross validation and widely applicable information criterion in singular learning theory . Journal of Machine Learning Research , 11 , 3571 – 3594 . 10.1109/scis-isis.2012.6505025

[b93] Wechsler , D. , & Petermann , F. ( 2012 ). Wechsler Adult Intelligence Scale—Fourth Edition . APA PsycTests. 10.1037/t15169-000

[b95] Wiecki , T. V. , Sofer , I. , & Frank , M. J. ( 2013 ). HDDM: Hierarchical Bayesian estimation of the drift-diffusion model in Python . Frontiers in Neuroinformatics , 7 , 14 . 10.1016/J.NEUROIMAGE.2020.117058 23935581 PMC3731670

[b101] Xu , W. , Kolozsvari , O. B. , Oostenveld , R. , & Hämäläinen , J. A. ( 2020 ). Rapid changes in brain activity during learning of grapheme-phoneme associations in adults . NeuroImage , 220 , 117058 . 10.1016/J.NEUROIMAGE.2020.117058 32561476

[b96] Xue , G. , Chen , C. , Jin , Z. , & Dong , Q. ( 2006 ). Language experience shapes fusiform activation when processing a logographic artificial language: An fMRI training study . NeuroImage , 31 ( 3 ), 1315 – 1326 . 10.1016/j.neuroimage.2005.11.055 16644241

[b97] Yarkoni , T. , Poldrack , R. A. , Nichols , T. E. , Van Essen , D. C. , & Wager , T. D. ( 2011 ). Large-scale automated synthesis of human functional neuroimaging data . Nature Methods , 8 ( 8 ), 665 – 670 . 10.1038/nmeth.1635 21706013 PMC3146590

[b98] Žarić , G. , Fraga-González , G. , Tijms , J. , Van der Molen , M. W. , Blomert , L. , & Bonte , M. ( 2015 ). Crossmodal deficit in dyslexic children: Practice affects the neural timing of letter-speech sound integration . Frontiers in Human Neuroscience , 9 ( June ), 1 – 14 . 10.3389/fnhum.2015.00369 26157382 PMC4478392

[b99] Zeguers , M. H. T. , Snellings , P. , Tijms , J. , Weeda , W. D. , Tamboer , P. , Bexkens , A. , & Huizenga , H. M. ( 2011 ). Specifying theories of developmental dyslexia: A diffusion model analysis of word recognition . Developmental Science , 14 ( 6 ), 1340 – 1354 . 10.1111/j.1467-7687.2011.01091.x 22010894

